# Bax Exists in a Dynamic Equilibrium between the Cytosol and Mitochondria to Control Apoptotic Priming

**DOI:** 10.1016/j.molcel.2012.12.022

**Published:** 2013-03-07

**Authors:** Barbara Schellenberg, Pengbo Wang, James A. Keeble, Ricardo Rodriguez-Enriquez, Scott Walker, Thomas W. Owens, Fiona Foster, Jolanta Tanianis-Hughes, Keith Brennan, Charles H. Streuli, Andrew P. Gilmore

**Affiliations:** 1 Wellcome Trust Centre for Cell-Matrix Research, Faculty of Life Sciences, University of Manchester, Manchester M13 9PT, UK

## Abstract

The proapoptotic Bcl-2 protein Bax is predominantly found in the cytosol of nonapoptotic cells and is commonly thought to translocate to mitochondria following an apoptotic stimulus. The current model for Bax activation is that BH3 proteins bind to cytosolic Bax, initiating mitochondrial targeting and outer-membrane permeabilization. Here, we challenge this and show that Bax is constitutively targeted to mitochondria but in nonapoptotic cells is constantly translocated back to the cytosol. Using live-cell spinning-disk confocal imaging with a combination of FLIP, FRAP, and photoactivatable GFP-Bax, we demonstrate that disrupting adhesion-dependent survival signals slows the rate of Bax’s dissociation from mitochondria, leading to its accumulation on the outer mitochondrial membrane. The overall accumulation of mitochondrial Bax following loss of survival signaling sensitizes cells to proapoptotic BH3 proteins. Our findings show that Bax is normally in a dynamic equilibrium between cytosol and mitochondria, enabling fluctuations in survival signals to finely adjust apoptotic sensitivity.

## Graphical abstract

**Graphical Abstract:**

**Figure:**
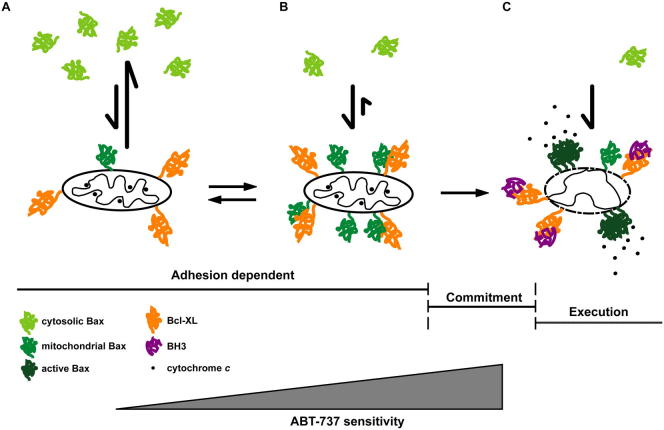

**Highlights:**

► Bax exists in equilibrium between the cytosol and mitochondria ► The retrotranslocation of Bax is independent of direct-activator BH3 proteins ► Changes in survival signaling modulate the dissociation rate of mitochondrial Bax ► Accumulation of Bax on mitochondria sensitizes cells to apoptosis

## Introduction

Activation of the intrinsic pathway of apoptosis leads to mitochondrial outer membrane permeabilization (MOMP). MOMP results in the release of soluble proteins from within the intermembrane space, activation of caspases, and cell death and represents the absolute point of no return within the intrinsic apoptotic pathway. MOMP is regulated by interactions between the Bcl-2 family of proteins, which present a possible point for therapeutic intervention in cancer (Youle and Strasser, 2008). Vertebrates have three distinct classes of Bcl-2 proteins: antiapoptotic proteins, including Bcl-XL, that suppress MOMP; the proapoptotic proteins Bax and Bak that activate MOMP; and the BH3 proteins that modulate the activities of the other two groups. Intrinsic apoptotic signaling converges on Bax and Bak, either of which is sufficient for driving MOMP in the majority of cells. Mice doubly deficient for Bax and Bak have profound developmental defects, and cells isolated from them are resistant to apoptotic stimuli that activate the mitochondrial pathway (Lindsten et al., 2000; Wei et al., 2001).

Both Bax and Bak are directed to the outer mitochondrial membrane (OMM) by a C-terminal tail anchor (TA) (Youle and Strasser, 2008). TA proteins are predominantly associated with the endoplasmic reticulum or mitochondria and are posttranslationally directed to their target organelle, where they are inserted via a single membrane span (Borgese et al., 2007). Bak, like the majority of TA proteins, including antiapoptotic Bcl-2 family members, is constitutively bound to its target membrane. However, unlike Bak, Bax is not normally found in mitochondria (Hsu and Youle, 1998). Instead, Bax is predominantly a monomer within the cytosol of nonapoptotic cells, where its TA sequence is folded back and hidden within a hydrophobic groove on the protein’s surface (Suzuki et al., 2000). Bax is thought to be converted into an active conformation that is capable of being inserted into mitochondria by interaction with BH3 proteins, such as Bim (Walensky and Gavathiotis, 2011). The majority of models for Bax activation involve the binding of a BH3 domain to Bax, displacing the hidden TA sequence through an induced conformational change, thus initiating Bax mitochondrial targeting and MOMP.

However, Bax targeting to mitochondria may be more complicated than these models suggest, because Bax can only interact with BH3 proteins if both are associated with membranes (Lovell et al., 2008). This implies that mitochondrial targeting of Bax is a prerequisite for its subsequent activation by BH3 domain binding. Furthermore, mitochondrial Bax may be removed back to the cytosol through its interaction with antiapoptotic proteins such as Bcl-XL (Edlich et al., 2011). Similarly, the localization of Bax to mitochondria following the loss of adhesion-dependent survival signals is reversed when cells reattach to the extracellular matrix (ECM) (Gilmore et al., 2000). Together, these studies suggest that mitochondrial targeting of Bax is not the terminal step in its activation driven by BH3 proteins.

Here, we show using live-cell spinning-disk confocal microscopy that Bax translocation between cytosol and mitochondria is a constitutive and dynamic process. By examining the dynamics of Bax’s association with mitochondria, we reveal that survival signals control the rate of Bax mitochondrial dissociation rather than its mitochondrial targeting. Reduced survival signals cause an overall accumulation of Bax on the OMM, where BH3 proteins can subsequently activate it. This new model explains how fluctuations in survival signals modulate apoptotic sensitivity, because cells dynamically adjust the concentration of multidomain proapoptotic Bcl-2 proteins on the OMM, which in turn fine-tunes their response to BH3 proteins.

## Results

### Bax Is in a Dynamic Equilibrium between the Cytosol and Mitochondria

In healthy cells, Bax is not exclusively cytosolic; rather, it is distributed between the cytosol and the mitochondria (Gilmore et al., 2000). The relative proportion of Bax in each fraction varies according to the level of stress and survival signaling (Valentijn et al., 2008b). This suggests that Bax exists in equilibrium between these two subcellular compartments. To test this hypothesis directly, we used spinning-disk confocal imaging to examine the dynamics of Bax subcellular localization in live cells.

**Figure 1:**
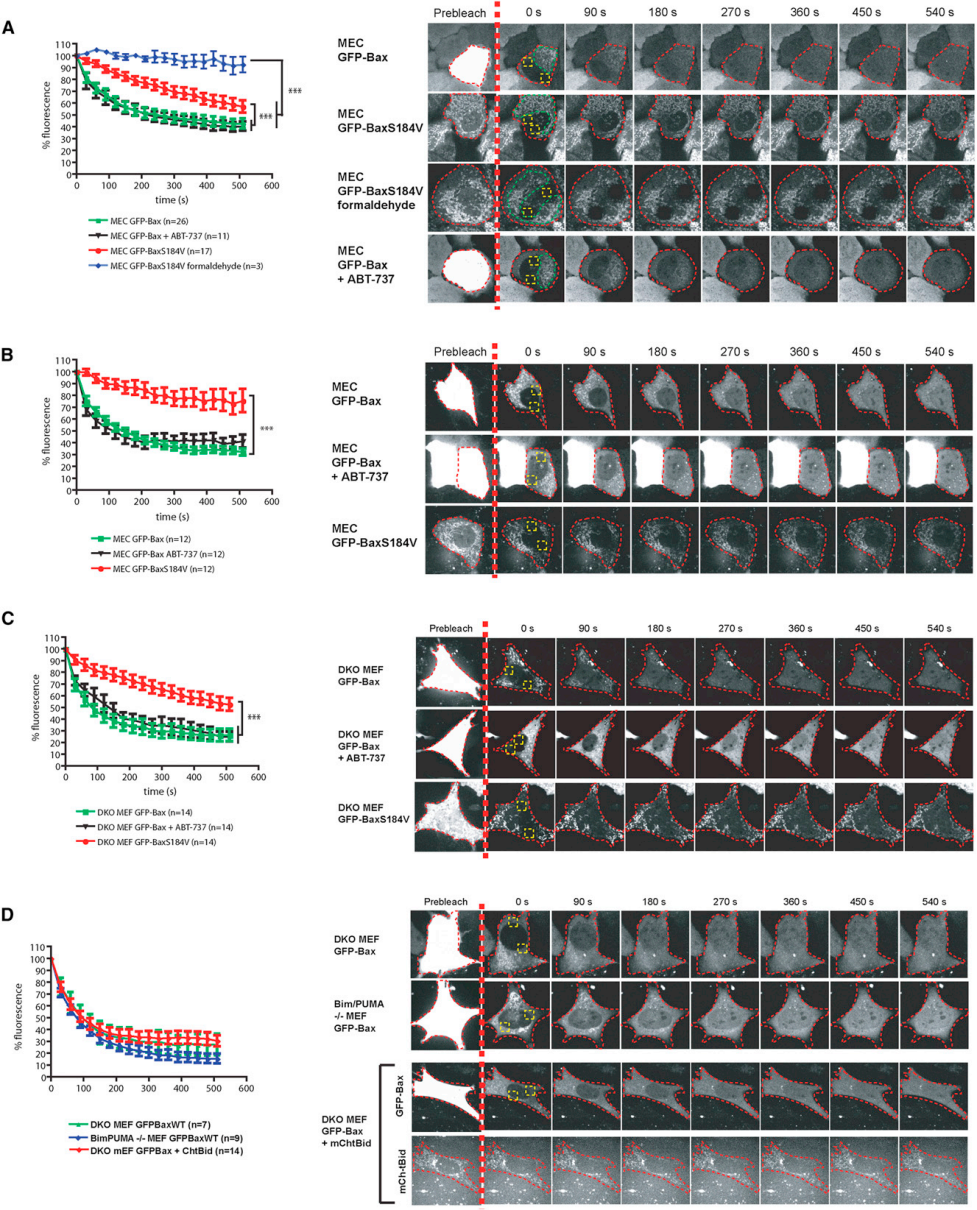
Bax Constantly Dissociates from Mitochondria in Nonapoptotic Cells (A) FLIP analysis of GFP-Bax and GFP-BaxS184V stably expressed in MECs. The cytosolic fractions of GFP-Bax and GFP-BaxS184V were photobleached in the yellow ROI. The red line indicates the outline of the imaged cell. Dissociation of the mitochondrial GFP was followed within the green ROIs over 10 min. The signals obtained post bleaching were analyzed as explained in the Experimental Procedures and normalized to 100% fluorescence. For all FLIP data, the first image post bleaching is set at 0 s, n = number of cells analyzed per condition, and error bars represent SEM. Data were analyzed by ANOVA. ^∗^ = p < 0.05; ^∗∗^ = p < 0.01; ^∗∗∗^ = p < 0.0001. Data indicate that GFP-Bax has a dissociation rate with t_1/2_ ≈ 70 s, which is unaffected by ABT-737. GFP-BaxS184V has a significantly reduced dissociation rate compared with GFP-Bax. See Movie S1. (B) FLIP analysis carried out as in (A) on MECs transiently expressing GFP-Bax or GFP-BaxS184V. Error bars represent SEM. (C) FLIP analysis of *Bax*^−*/*−^*Bak*^−*/*−^ DKO MEFs transiently expressing GFP-Bax or GFP-BaxS184V. See Movie S2. Error bars represent SEM. (D) FLIP analysis of GFP-Bax expressed in either *Bax*^−*/*−^*Bak*^−*/*−^ or *Bim*^−*/*−^*PUMA*^−*/*−^ DKO MEFs. In the lower panel, *Bax*^−*/*−^*Bak*^−*/*−^ DKO MEFs expressed both GFP-Bax and mCherry-tBid. See Movie S3. Error bars represent SEM.

Mouse mammary epithelial cells (MECs) that stably expressed GFP-Bax were generated. To examine Bax dissociation from mitochondria in healthy adherent cells, we used fluorescence loss in photobleaching (FLIP). By photobleaching two defined regions of interest (ROIs, indicated in yellow, Figure 1A), we were able to bleach almost all of the mobile, cytosolic fraction of GFP-Bax (as it would pass through either ROI during bleaching), thus allowing visualization of the bound mitochondrial fraction outside of these ROIs (Figure 1A; Movie S1 available online). Images were then captured every 30 s, and mitochondrial GFP-Bax was quantified within a third ROI (indicated in green). Mitochondrial GFP-Bax within the green ROI trafficked to the cytosol, showing exponential decay from mitochondria (half-life [t_1/2_] ≈ 70 s). Similar results were obtained in transient transfections, wherein GFP-Bax was expressed at higher levels (Figure 1B). In contrast, there was no significant mitochondrial dissociation of GFP-Bak over this time period (Figure S1A).

To determine whether the dissociating GFP-Bax is loosely associated with mitochondria or embedded within the OMM, we isolated mitochondria from adherent cells and extracted them with sodium carbonate. The majority of mitochondrial GFP-Bax was resistant to sodium carbonate, implying that GFP-Bax seen dissociating from mitochondria had been at least partially inserted into the membrane (Figure S1B; Valentijn et al., 2008a). To test this further, we analyzed the dynamics of GFP-BaxS184V, which contains a substitution within the TA resulting in constitutive mitochondrial localization via α helix 9 (Nechushtan et al., 1999) (Figures 1A and 1B; Movie S1). When analyzed by sodium carbonate extraction, all of the mitochondrial GFP-BaxS184V was membrane inserted (Figure S1B). Surprisingly, GFP-BaxS184V also dissociated from mitochondria, but the dissociation rate (t_1/2_ ≈ 600 s) was nearly ten times slower than that of GFP-Bax. As a control, FLIP analysis of cells expressing GFP-BaxS184V fixed in 4% formaldehyde discounted that any significant photobleaching occurred during image acquisition (Figure 1A; Movie S1). We also carried out FLIP analysis of the Bax TA alone, tagged to YFP (YFP-BaxTMD; the C-terminal 23 amino acids [aa] of Bax). The 23 aa TA of Bax has been shown, again by carbonate extraction, to insert into the OMM (Schinzel et al., 2004; Valentijn et al., 2008a). We found that YFP-BaxTMD rapidly redistributed throughout the cell (Figure S1C), with a loss of fluorescence within the nonphotobleached areas and a concomitant fluorescence recovery within the bleached ROIs (t_1/2_ for recovery ≈ 30–40 s). These data show that although Bax and its constitutive mitochondrial mutants target to mitochondria via TA membrane insertion, they are constantly being extracted from the OMM back to the cytosol.

To determine whether antiapoptotic proteins are required for the dissociation of mitochondrial Bax, we performed FLIP analysis in the presence of 5 μM ABT-737, a BH3 mimetic that inhibits Bcl-2 and Bcl-XL (Oltersdorf et al., 2005). ABT-737 had no effect on the dissociation of GFP-Bax from mitochondria (Figures 1A and 1B; Movie S1). Moreover, endogenous Bax and Bak did not influence the dissociation rate of GFP-Bax, seen in *Bax*^−*/*−^*Bak*^−*/*−^ double-knockout mouse embryonic fibroblasts (DKO MEFs) transiently expressing GFP-Bax or GFP-BaxS184V (Figure 1C; Movie S2). To address whether Bax dynamics required direct-activator BH3 proteins, we performed FLIP on GFP-Bax expressed in *Bim*^−*/*−^*PUMA*^−*/*−^ DKO MEFs (Figure 1D; Movie S3). There was no significant difference in the GFP-Bax dissociation rate in these cells. Furthermore, we were unable to detect cleaved Bid in *Bim*^−*/*−^*PUMA*^−*/*−^ DKO MEFs (Figure S1D), suggesting that they lack all functional direct-activator BH3 proteins. To test directly whether Bid influenced Bax dissociation, we performed FLIP in *Bax*^−*/*−^*Bak*^−*/*−^ DKO MEFs coexpressing GFP-Bax and mCherry-tBid. We observed two phenotypes. One population showed Bax dissociation with identical kinetics as in all the other cells analyzed (Figure 1D; Movie S3). The second population of cells all underwent apoptosis within 10 min following photobleaching and appeared to have punctate spots of GFP-Bax that remained throughout the movie (Figure S1E). These results suggest that Bax dissociation from the OMM occurs independently of Bcl-2 survival proteins and direct-activator BH3 proteins.

**Figure 2:**
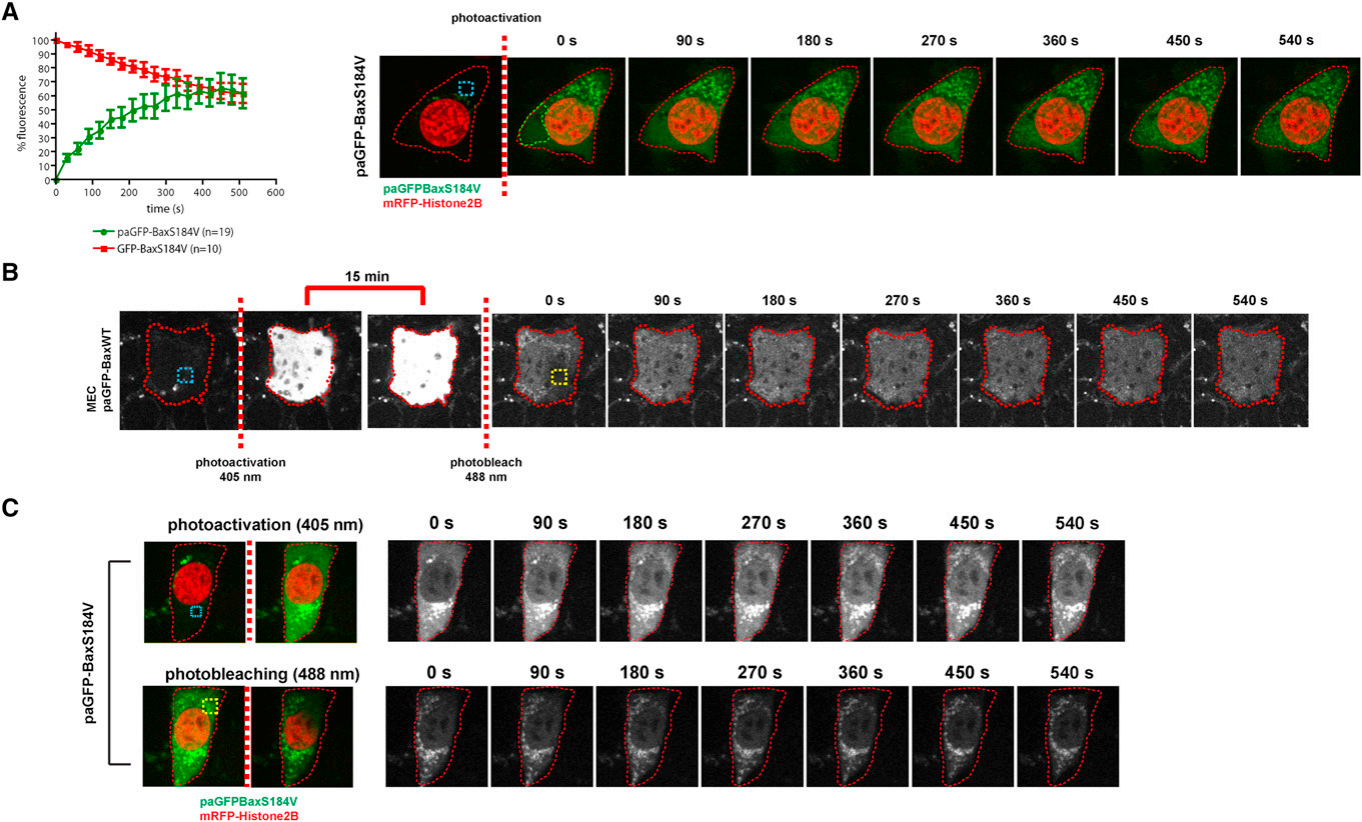
Bax Exists in a Dynamic Equilibrium between Mitochondria and the Cytosol (A) MECs transiently expressing paGFP-BaxS184V were photoactivated at 405 nm in the blue ROI. Association of paGFP-BaxS184V on mitochondria was monitored over 10 min within the green ROI. mRFP-H2B was coexpressed from the same plasmid as paGFPBaxS184V to allow identification of cells for photoactivation. See Movie S4. Error bars represent SEM. (B) MECs transiently expressing paGFP-Bax were photoactivated as in (A). Fifteen minutes post activation, the same ROI was photobleached at 488 nm. Cells were imaged for 10 min. See Movie S5. (C) paGFP-BaxS184V was photoactivated at 405 nm within the blue ROI, and its accumulation on mitochondria was imaged as in (A). FLIP was then performed by photobleaching within the yellow ROI, and redistribution to the bleached area followed. mRFP-H2B is shown in red.

To compare the mitochondrial association of Bax with dissociation, we expressed Bax and BaxS184V tagged with photoactivatable GFP (paGFP-Bax and paGFP-BaxS184V) (Patterson and Lippincott-Schwartz, 2002). Because Bax-S184V showed a slow dissociation rate, we reasoned that after photoactivation in one ROI (Figure 2A, blue ROI, Movie S4), we could measure mitochondrial association in a distinct ROI (Figure 2A, green ROI). Following photoactivation at 405 nm, paGFP-BaxS184V associated with mitochondria with a t_1/2_ of ∼200 s. The different association and dissociation (600 s) rates of BaxS184V indicate that the equilibrium favors a mitochondrial distribution. In contrast, a similar association rate for paGFP-Bax (i.e., to BaxS184V) would favor a cytosolic localization due to its faster dissociation rate (70 s). We were unable to measure the association rate of paGFP-Bax due to its predominantly cytosolic distribution. Instead, we carried out photoactivation at 405 nm, followed after 15 min by photobleaching at 488 nm in the same ROI (Figure 2B; Movie S5). This revealed that paGFP-Bax associated with mitochondria within 15 min and then dissociated at a similar rate as GFP-Bax.

For Bax to be in cytosolic-mitochondrial equilibrium, we would expect that Bax molecules dissociating to the cytosol would remain functional and thus able to reassociate with mitochondria. To test this, we photoactivated paGFP-BaxS184V within a defined ROI (marked in blue, Figure 2C) and then performed fluorescence recovery after photobleaching (FRAP) once it had associated with mitochondria by photobleaching within the yellow ROI. This bleached both the mitochondrial paGFP-BaxS184V within the yellow ROI and cytosolic paGFP-BaxS184V. Thus, any GFP that subsequently associated with mitochondria within the yellow ROI would have first dissociated from unbleached mitochondria elsewhere in the cell. We found that fluorescent paGFP-BaxS184V reassociated within the bleached area, indicating that Bax is still functional after mitochondrial dissociation.

Together, these results demonstrate that Bax molecules traffic between the cytosol and mitochondria and that in healthy cells Bax is in dynamic equilibrium between these two cellular compartments.

### Bax and Bcl-XL Stabilize the Membrane-Associated Forms of Each Other

**Figure 3:**
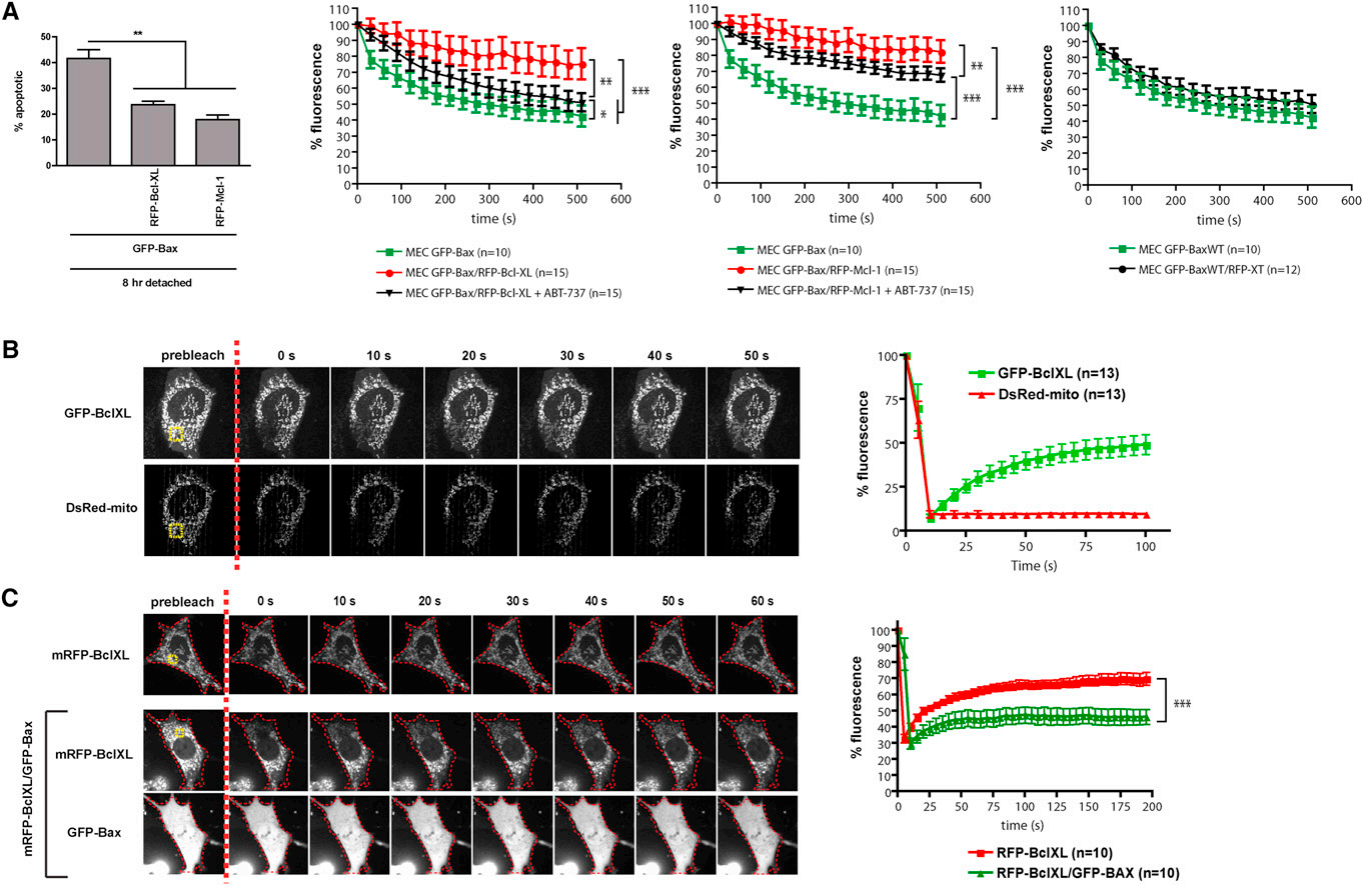
Bax and Bcl-XL Can Stabilize Each Other on Mitochondria (A) MECs stably expressing GFP-Bax were transfected with expression vectors for either mRFP-Bcl-XL or mRFP-Mcl-1. Eighteen hours post transfection, FLIP analysis was carried out as in Figure 1A in the presence or absence of ABT-737. The three graphs shown are from the same experiment, and the data for GFP-Bax (green line) are the same in all three plots. The bar graph on the left indicates that mRFP-Bcl-XL and mRFP-Mcl-1 inhibited detachment-induced apoptosis. Error bars represent SEM. See Figure S2. (B) FRAP analysis of Bcl-XL and DsRed-Mito. MECs transiently expressing GFP-BclXL and DsRed-Mito were photobleached in the yellow ROI and imaged every 5 s. Fluorescence intensity was analyzed within the ROI and normalized to 100%. Error bars represent SEM. See Movie S6. (C) *Bax*^−*/*−^*Bak*^−*/*−^ DKO MEFs transiently expressing mRFP-Bcl-XL, either alone or with GFP-Bax, were analyzed by FRAP as in (B). Error bars represent SEM. See Movie S7.

A recent study indicated that Bcl-XL or Mcl-1 increased retrotranslocation of Bax from mitochondria to the cytosol (Edlich et al., 2011). However, our data using ABT-737 suggested that inhibiting Bcl-XL or Bcl-2 did not affect the rate of Bax dissociation from mitochondria. To investigate this further, we overexpressed mRFP-BclXL or mRFP-Mcl-1 in MECs stably expressing GFP-Bax. This inhibited apoptosis and reduced the dissociation rate of mitochondrial GFP-Bax (Figure 3A; Figure S2). This was not seen in cells that expressed just mRFP targeted to the OMM via the Bcl-XL TA sequence (mRFP-XT), suggesting that Bcl-XL and Mcl-1 retained some Bax in an inactive state on mitochondria. The reduction in GFP-Bax dissociation in mRFP-Bcl-XL-expressing cells was reversed by ABT-737. In contrast, ABT-737 caused a minor increase in GFP-Bax dissociation in cells expressing mRFP-Mcl-1. Thus, in MECs, Bcl-XL stabilizes mitochondrial-associated Bax.

Bcl-XL was also in equilibrium between the cytosol and mitochondria. FRAP studies on cells coexpressing GFP-Bcl-XL and DsRed-mito (Figure 3B; Movie S6) revealed that GFP-BclXL recovered to about 50% of its initial fluorescence. Thus, about half of the protein was stably associated with the OMM. The other half, representing the mobile fraction, recovered with a t_1/2_ ≈ 20 s. There was no recovery of DsRed-mito in this time frame, indicating GFP-Bcl-XL recovery was not due to mitochondrial dynamics. If Bcl-XL expression reduced the dissociation of GFP-Bax through an interaction between them, then it might be expected that Bax would similarly stabilize mitochondrial Bcl-XL. To test this, we performed FRAP on Bcl-XL in the presence or absence of Bax (Figure 3C; Movie S7). mRFP-BclXL expressed in *Bax*^−*/*−^*Bak*^−*/*−^ DKO MEFs showed similar FRAP kinetics as GFP-BclXL in MECs (∼50% recovery; t_1/2_ ≈ 20 s). Coexpression of GFP-Bax significantly decreased the mobile fraction of Bcl-XL, suggesting that increasing the amount of Bax stabilizes a fraction of the total cellular Bcl-XL on the OMM. Thus there is a costabilization of Bax and Bcl-XL on the mitochondrial membrane.

### Adhesion-Mediated Changes in Survival Signals Alter the Equilibrium between Cytosolic and Mitochondrial Bax via Its Dissociation Rate

**Figure 4:**
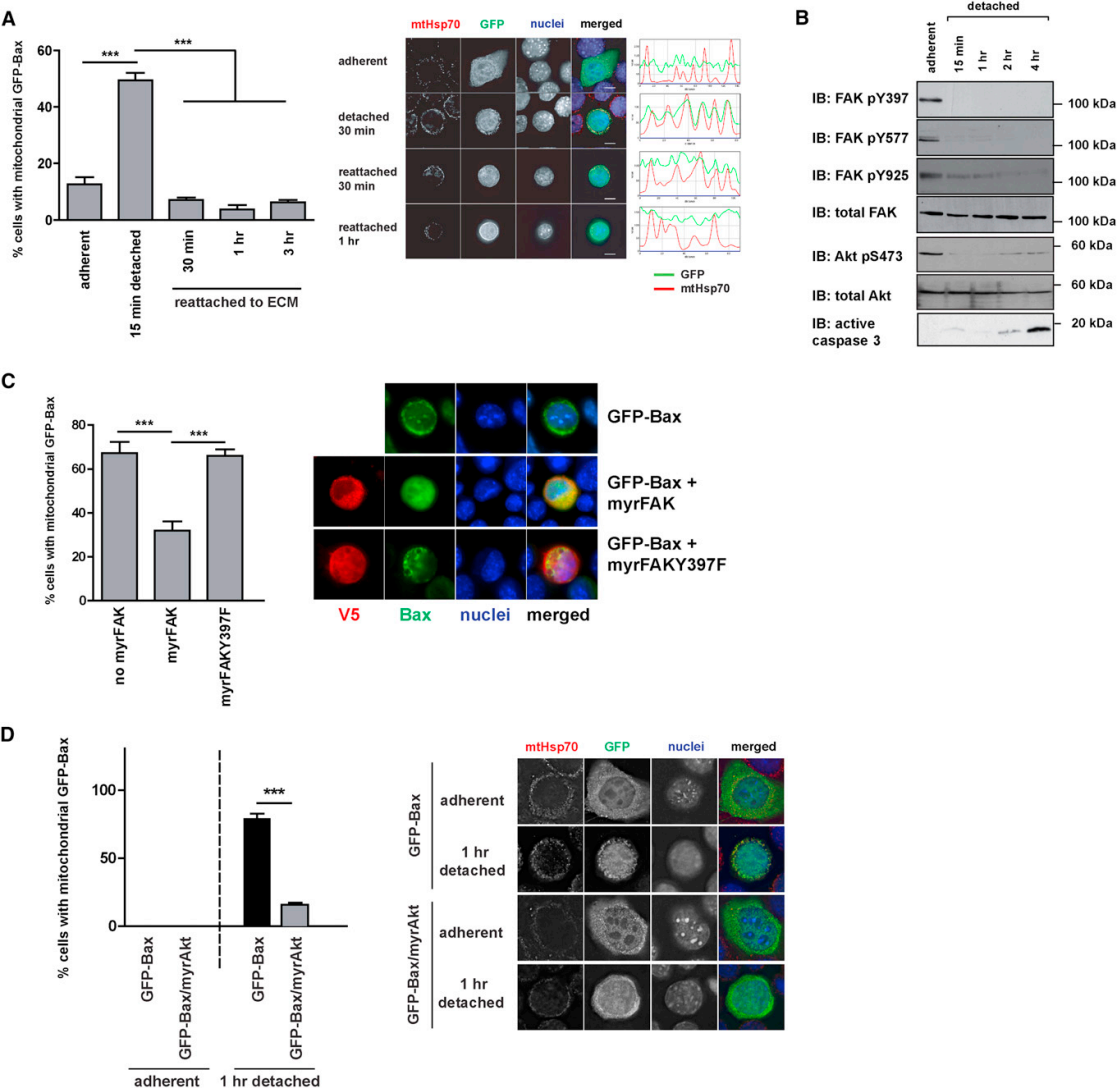
Detachment of MECs Followed by Reattachment Rapidly Alters the Subcellular Equilibrium of Bax (A) MECs transiently expressing GFP-Bax were left adherent, detached for 30 min, or detached for 30 min before reattaching to ECM for the indicated times. After fixation, cells were immunostained for anti-GFP and anti-mtHsp70. The number of cells displaying predominantly mitochondrial GFP-Bax was quantified. Error bars represent SEM. Data was analyzed by ANOVA; ^∗∗∗^ = p < 0.0001. Representative images and line profiles of each image indicating coincidence of GFP and mtHsp70 are shown on the right. Scale bar represents 10 μm. See also Figure S3A. (B) Detachment of MECs leads to a rapid loss of FAK and Akt activity. Adherent cells and cells detached for various times were immunoblotted (IB) for anti-FAK pY397, pY577, pY925, and anti-total FAK (left panel) or for anti-Akt pS473, anti-Akt pT308, anti-total Akt, and anti-active caspase 3 (right panel). (C) MECs were transfected with pEGFP-Bax either alone or in combination with pCDNA6-myrFAK or the inactive variant myrFAKY397F. Eighteen hours post transfection, cells were detached for 15 min, cytospun on polysine slides, and immunostained for anti-GFP and anti-V5 (for detection of coexpressed myrFAK). The number of V5-positive cells with predominantly mitochondrial GFP-Bax was determined. Data represent the mean of three independent experiments. Error bars represent SEM. Data was analyzed by ANOVA; ^∗∗^ = p < 0.01. ^∗∗∗^; = p < 0.0001. The panel on the right shows representative images. (D) MECs were transfected with either pCDH-GFPBax or pCDH-myrAkt-GFPBax. Eighteen hours post transfection, cells were left adherent or detached for 1 hr. Cells were immunostained for anti-GFP and anti-mtHsp70. The number of cells with predominantly mitochondrial GFP-Bax was determined. The mean of three independent experiments is shown. Error bars represent SEM. Data were analyzed by ANOVA; ^∗∗∗^ = p < 0.0001. The panel on the right shows representative images. See also Figure S3B.

Given that GFP-Bax exists in cytosolic-mitochondrial equilibrium, our results suggest that Bax translocation following an apoptotic signal might be explained by reduced dissociation from mitochondria rather than induced association. To test this hypothesis, we used anoikis to examine the dynamics of Bax distribution. In this model, detaching MECs from the ECM induces a rapid (within 15 min) accumulation of Bax on mitochondria, which can be reversed if cells reattach to the ECM prior to MOMP (Figure 4A; Figure S3A) (Gilmore et al., 2000). Importantly, Bax translocation and MOMP are temporally distinct in MEC anoikis, because death requires prolonged loss of cell adhesion (>8 hr) (Valentijn et al., 2003).

Focal adhesion kinase (FAK) is a nonreceptor tyrosine kinase that is recruited to sites of integrin-mediated adhesion, where it is activated for suppression of anoikis (Schaller, 2010; Zouq et al., 2009). Within 15 min of detaching cells from the ECM, endogenous FAK became dephosphorylated (on residues Y397, Y577, and Y925) with the same kinetics as Bax accumulation on the OMM (Figures 4A and 4B; Figure S3A). In contrast, a FAK variant that remained active in detached cells (myrFAK; Zouq et al., 2009) maintained GFP-Bax in the cytosol, whereas an inactive mutant FAK (myrFAKY397F) did not (Figure 4C). Similarly, Akt, which is a downstream effector of FAK-dependent survival, was inactivated within 15 min of detachment (Figure 4B). Constitutively active Akt (myrAkt) also inhibited GFP-Bax accumulation on mitochondria (Figure 4D; Figure S3B).

**Figure 5:**
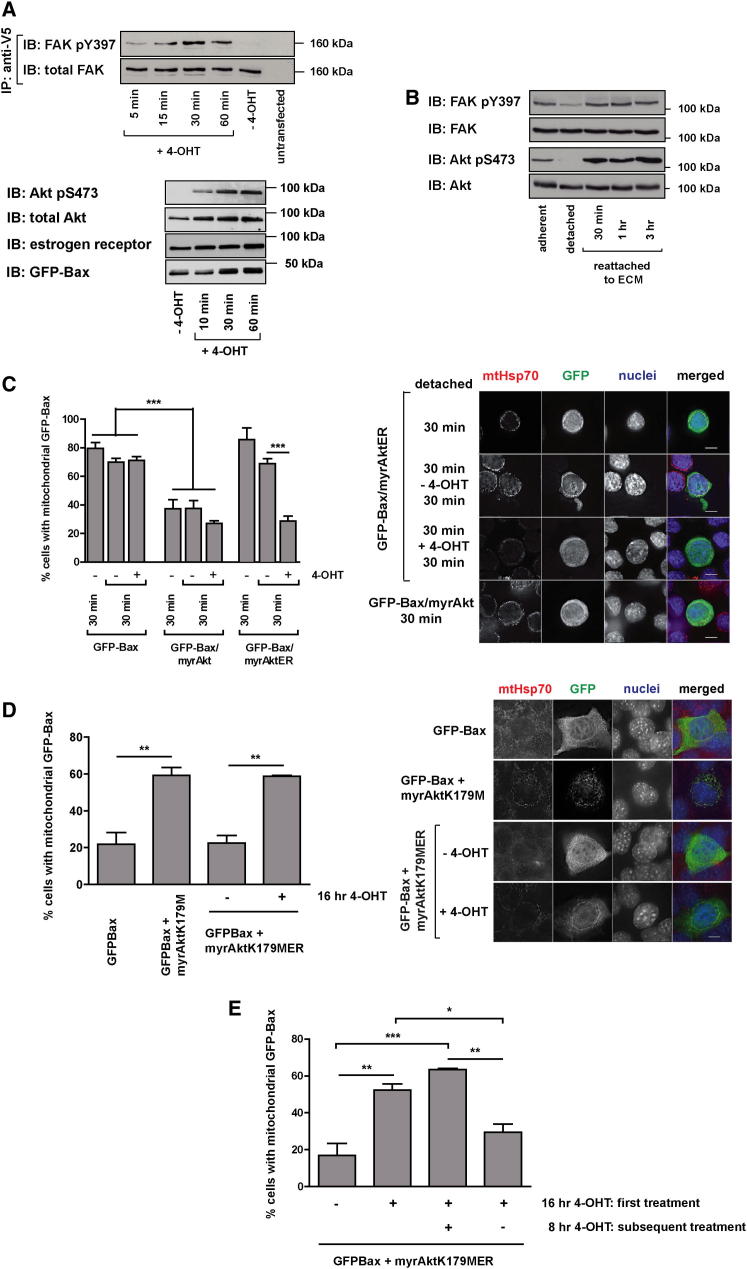
Activation of FAK and Akt Leads to the Dissociation of Mitochondrial Bax in Detached Cells (A) MECs transiently expressing myrFAKER (upper panel) or myrAktER (lower panel) were treated with 4-OHT for the indicated times. Lysates were immunoblotted with the indicated antibodies. IP, immunoprecipitation. (B) Reattachment of MECs leads to the rapid activation of FAK and Akt. MECs were analyzed by immunoblotting for phosphorylation of FAK on tyrosine 397 and Akt on serine 473. (C) MECs transiently expressing GFP-Bax alone or in combination with myrAktER or myrAkt (expressed from the same plasmid) were detached for 30 min. Cells were left untreated for an additional 30 min or treated with 4-OHT for 30 min, then immunostained for anti-GFP and anti-mtHsp70. The proportion of cells with predominantly mitochondrial GFP-Bax was quantified. The mean of three independent experiments is shown. Error bars represent SEM. Data were analyzed by ANOVA; ^∗∗∗^ = p < 0.0001. The panel on the right shows representative images. (D) Adherent MECs coexpressing GFP-Bax and either myrAktK179M or myrAktK179MER were untreated or treated with 4-OHT. The distribution of GFP-Bax was determined as in (C). The mean of three independent experiments is shown. Error bars represent SEM. Data were analyzed by ANOVA; ^∗∗^ = p < 0.01. Representative images are shown on the right. (E) MECs expressing GFP-Bax and myrAktK179MER were left untreated or treated with 4-OHT, followed by 8 hr either with or without washing out the 4-OHT. The subcellular distribution of GFP-Bax was then quantified as in (C). Error bars represent SEM.

To determine whether FAK and Akt signaling are sufficient to cause the dissociation of mitochondrial Bax, we expressed estrogen receptor (ER) fusion proteins, myrFAKER (Figure S4) and myrAktER (Kohn et al., 1998), that are activated by the estrogen analog, 4-hydroxytamoxifen (4-OHT). The kinetics of 4-OHT-induced activation of myrFAKER and myrAktER in detached MECs (Figure 5A) were similar to the activation of endogenous FAK and Akt during reattachment to the ECM (Figure 5B). Pretreatment of cells expressing myrFAKER or myrAktER with 4-OHT prior to detaching them from the ECM inhibited mitochondrial accumulation of GFP-Bax to the same extent as seen with the constitutively active form of each kinase (Figures S5A and S5B). Moreover, activation of myrFAKER (Figure S5C) and myrAktER (Figure 5C) in detached MECs induced GFP-Bax dissociation from mitochondria in a similar manner to that seen after ECM reattachment.

To confirm the role of adhesion signaling on Bax subcellular distribution, we expressed a kinase-dead variant of Akt, myrAktK179M, in adherent, spread MECs. This induced mitochondrial accumulation of GFP-Bax (Figure 5D), as did myrAktK179MER following 4-OHT treatment. Furthermore, washing out the 4-OHT for 8 hr reversed the accumulation of mitochondrial GFP-Bax induced by myrAktK179MER (Figure 5E). Thus, switching Akt signaling on and off reversibly shifts the equilibrium of GFP-Bax between the cytosolic and mitochondrial compartments.

**Figure 6:**
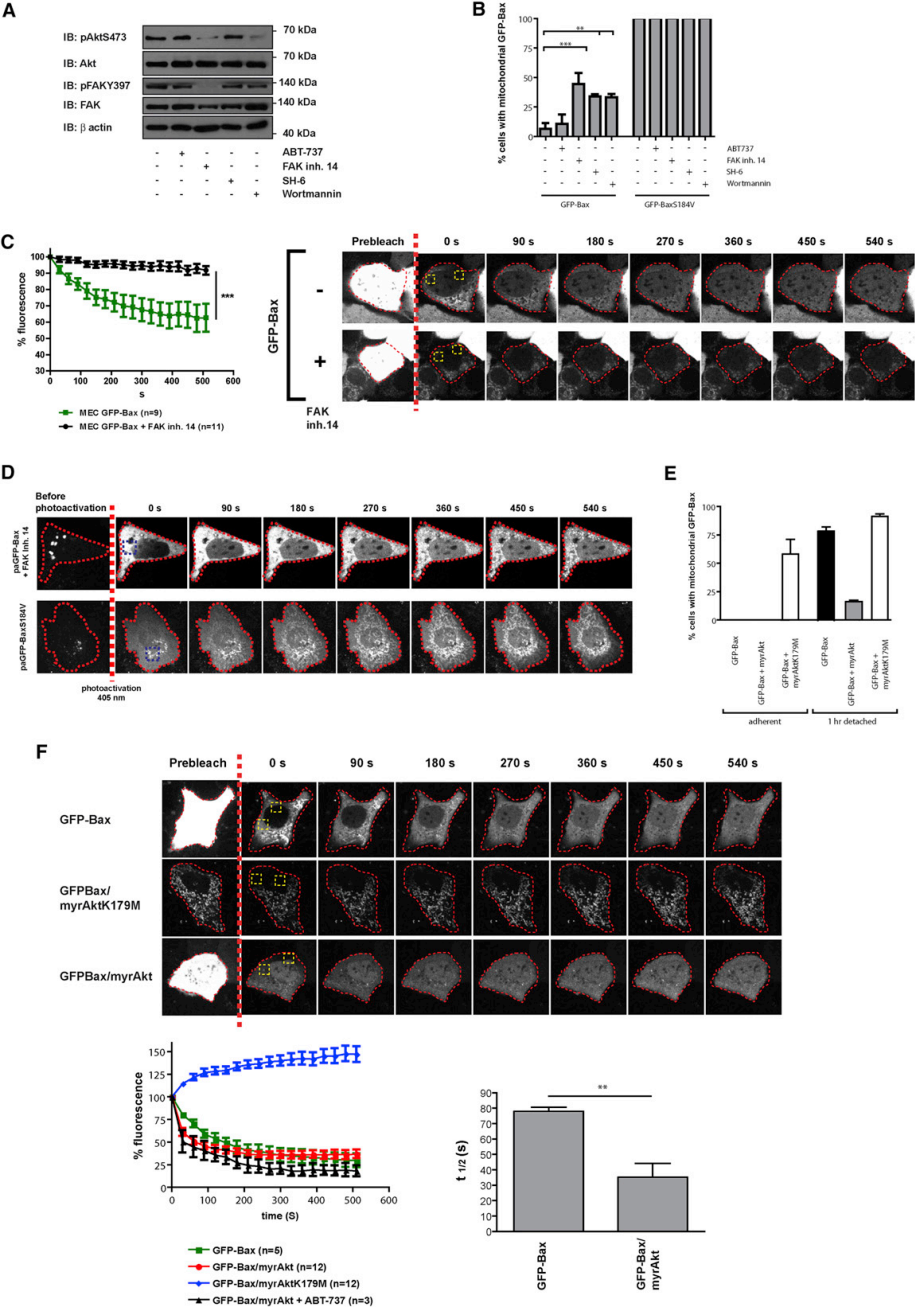
FAK and Akt Signaling Control Bax Mitochondrial Dissociation (A) Effect of the small-molecule inhibitors on the FAK-Akt signaling axis. Adherent MECs were treated with either ABT-737, FAK inhibitor 14, SH-6, or Wortmannin and immunoblotted for Akt pS473, total AKT, FAK pY397, total FAK, and β actin. (B) MECs expressing GFP-Bax or GFP-BaxS184V were treated with ABT-737, FAK inhibitor 14, SH-6, or Wortmannin. Cells were immunostained with anti-GFP and anti-mtHsp70, and the number of cells with predominantly mitochondrial Bax was quantified. Data represent the mean of three independent experiments. Error bars represent SEM. Data were analyzed by ANOVA; ^∗∗^ = p < 0.01; ^∗∗∗^ = p < 0.0001. (C) MECs stably expressing GFP-Bax were treated with DMSO or FAK inhibitor 14. FLIP was performed as in Figure 1, and the dissociation rate of mitochondrial Bax was quantified. See Movie S8. (D) MECs expressing paGFP-Bax were treated with FAK inhibitor 14, photoactivated (blue ROI), and imaged over 15 min. Untreated MECs expressing paGFP-BaxS184V are shown in the lower panel for comparison, before and after photoactivation. (E) MECs transiently expressing GFP-Bax alone or with myrAkt or myrAktK179M were left adherent or detached for 1 hr. The proportion of cells with mitochondrial Bax was quantified as before. Error bars represent SEM. (F) FLIP analysis on MECs expressing GFP-Bax alone or with either myrAkt or myrAktK179M. The t_1/2_ for dissociation of GFP-Bax was determined. ABT-737 (5 μM) had no effect on the dissociation of GFP-Bax in MECs coexpressing myrAkt. Error bars represent SEM. See Movie S9.

Next, we asked whether the FAK-Akt signaling axis acts directly on the dissociation rate of mitochondrial Bax. It was not possible to monitor GFP-Bax dynamics in detached cells, so we mimicked anoikis in adherent, spread cells by inhibiting FAK-Akt signaling using small-molecule inhibitors of FAK (FAK inh.14), PI3 kinase (Wortmannin), or Akt (SH-6) (Figure 6A). These inhibitors induced the mitochondrial association of GFP-Bax (Figure 6B). Moreover, FLIP analysis showed that treatment of MECs with FAK inh.14 almost completely abolished the dissociation of GFP-Bax from mitochondria (Figure 6C; Movie S8). To follow mitochondrial Bax association, cells expressing paGFP-Bax were treated with FAK inh.14, and the cytosolic paGFP-Bax was photoactivated (Figure 6D). The association of paGFP-Bax with mitochondria in the presence of FAK inh.14 occurred within a similar time frame to that seen with paGFP-BaxS184V (Figure 6D, lower panel). Thus, FAK is required for dissociation of mitochondrial Bax in MECs.

SH-6 and Wortmannin caused MECs to accumulate large vacuoles that obscured mitochondrial morphology in live-cell imaging. Therefore, we used the active myrAkt or kinase-dead myrAktK179M in adherent MECs, which inhibited detachment-induced GFP-Bax translocation and induced mitochondrial accumulation of GFP-Bax in adherent cells, respectively (Figure 6E). We measured GFP-Bax dissociation by FLIP in cells expressing either the kinase-dead or active forms of myrAkt (Figure 6F; Movie S9). myrAktK179M abolished GFP-Bax dissociation from mitochondria. In contrast, active myrAkt significantly increased the rate of GFP-Bax dissociation (t_1/2_ ≈ 40 s) compared to cells expressing GFP-Bax alone (t_1/2_ ≈ 80 s). ABT-737 had no effect on the dissociation rate of GFP-Bax coexpressed with myrAkt (Figure 6F).

Together, these data reveal that adhesion survival signals regulate the subcellular localization of Bax by influencing its dissociation from mitochondria into the cytosol.

### Alterations in the Rate of Bax Dissociation Adjust Cell Sensitivity to Sensitizer BH3 Proteins

**Figure 7:**
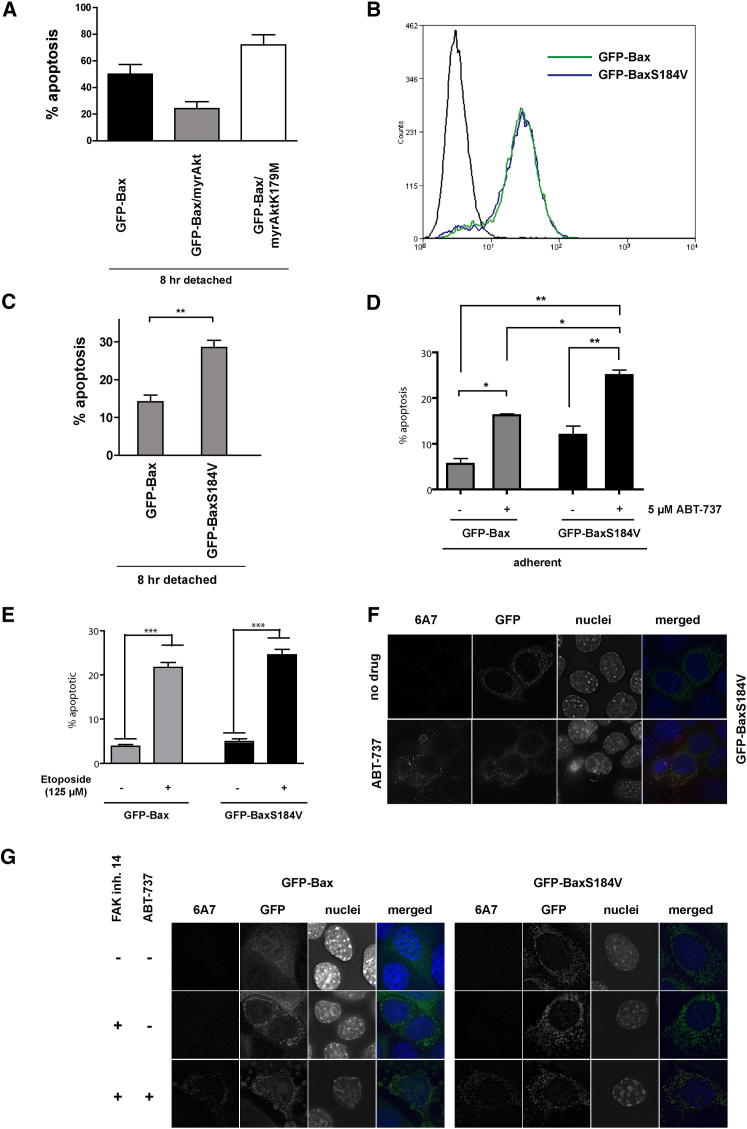
Inhibiting Mitochondrial Bax Dissociation Sensitizes Cells to BH3 Mimetics (A) MECs expressing GFP-Bax alone or with myrAkt or myrAktK179M were detached from the ECM for 8 hr, and apoptosis was quantified. Data represent the mean of three independent experiments. Error bars represent SEM. Data were analyzed by ANOVA; ^∗∗^ = p < 0.01. (B) Flow-cytometery analysis of GFP in MECs stably expressing GFP-Bax or GFP-BaxS184V. (C) MECs from (B) were detached from the ECM for 8 hr. Apoptosis was then quantified. Error bars represent SEM. Data were analyzed by ANOVA; ^∗∗^ = p < 0.01. (D) MECs from (B) were treated with 5 μM ABT-737 as indicated. Apoptosis was quantified. Data represent the mean of three independent experiments. Error bars represent SEM. Data were analyzed by ANOVA; ^∗^ = p < 0.05; ^∗∗^ = p < 0.01. (E) MECs from (B) were treated with 125 μM etopisode as indicated. Apoptosis was quantified. Data represent the mean of three independent experiments. Error bars represent SEM. Data were analyzed by ANOVA; ^∗∗∗^ = p < 0.0001. (F) Adherent MECs expressing GFP-BaxS184V were either left untreated or treated with 5 μM ABT-737. Cells were immunostained with anti-Bax monoclonal 6A7 and anti-GFP. Exposure and postcapture manipulation were identical for all images. (G) Adherent MECs stably expressing either GFP-Bax or GFP-BaxS184V were treated with FAK inhibitor either alone or in combination with ABT-737. Cells were immunostained as in (F).

The reversible changes in Bax distribution described above occurred prior to apoptosis. To determine whether Bax dynamics regulate apoptotic sensitivity, we measured death 8 hr after ECM detachment in cells transiently expressing GFP-Bax with or without myrAkt or myrAktK179M. The extent of apoptosis correlated with the ability of the different Akt constructs to either induce or inhibit Bax dissociation from mitochondria (Figure 7A).

To investigate the consequences of reduced Bax mitochondrial dissociation on apoptotic sensitivity following detachment from the ECM, we generated MECs that stably expressed equal levels of either GFP-Bax or GFP-BaxS184V (Figure 7B). The amount of apoptosis was significantly higher in cells expressing GFP-BaxS184V, which had reduced Bax dissociation from mitochondria, than in cells expressing GFP-Bax (Figure 7C). Moreover ABT-737 elevated apoptosis in both GFP-Bax- and GFP-BaxS184V-expressing adherent cells, but the extent of apoptosis enhancement was significantly greater in the cells where GFP-Bax mitochondrial dissociation was reduced (Figure 7D). Importantly, there was no difference in apoptosis between GFP-Bax and GFP-BaxS184V cells in controls treated for 24 hr with etoposide, a potent and irreversible apoptotic stimulus (Figure 7E). These results indicate that accumulated mitochondrial Bax is maintained in an inactive state at least in part by the action of antiapoptotic Bcl-2 and/or Bcl-XL. Furthermore, reducing the dissociation rate of Bax alone is sufficient to alter the sensitivity of cells to the activity of sensitizer BH3 mimetics.

Lastly, we examined Bax conformation using the monoclonal antibody 6A7, which recognizes N-terminal epitopes exposed during its activation. Cells expressing GFP-BaxS184V were negative for 6A7 staining, despite its predominantly mitochondrial distribution (Figure 7F). However, treatment with ABT-737 increased the number of 6A7-positive cells, all of which had apoptotic nuclei. Similarly, FAK inhibition alone, which prevented mitochondrial Bax dissociation, did not cause Bax N-terminal epitope exposure in adherent GFP-Bax or GFP-BaxS184V MECs (Figure 7G).

Together, these data reveal that the accumulation of Bax on mitochondria precedes N-terminal exposure associated with its activation. Furthermore, the N-terminal conformation change occurs downstream of reversible Bax mitochondrial association. The implication is that antiapoptotic Bcl-2 proteins hold Bax that has accumulated on mitochondria in an inactive state and that derepression of these antiapoptotic proteins contributes to Bax activation and cell death.

## Discussion

Many of the studies that have defined how interactions between Bcl-2 family proteins regulate apoptosis have been carried out in systems that lack the dynamic signal transduction occurring in live cells. Consequently, how these interactions are influenced in real time by changes in signaling is often not incorporated into existing models. In this paper, we have used live-cell imaging to examine the dynamic interactions of Bax with mitochondria. Our results show that “translocation” of Bax during apoptosis is actually a shift in its equilibrium between the cytosolic and membrane fractions. This occurs by reducing the dissociation rate of mitochondrial Bax when survival signals are inhibited, leading to its rapid accumulation on the OMM and a significant increase in apoptotic priming.

BH3 proteins are proposed to be the conduit through which apoptotic signaling occurs (Bouillet and Strasser, 2002) and are either direct activators of Bax and Bak or sensitizers that inhibit antiapoptotic Bcl-2 proteins (Chipuk et al., 2010). Our results show that the dynamics of GFP-Bax in *Bim*^−*/*−^*PUMA*^−*/*−^ DKO MEFs, or in *Bax*^−*/*−^*Bak*^−*/*−^ DKO MEFs expressing tBid, are identical to those seen in wild-type cells. Similarly, mimicking sensitizer BH3 proteins with ABT-737 did not alter Bax mitochondrial dissociation or result in accumulation of GFP-Bax on mitochondria. Thus, the fundamental process of Bax membrane association and retrotranslocation does not require its activation by BH3 proteins. This is significant because many models of mitochondrial apoptosis place BH3 proteins upstream of Bax translocation (Gavathiotis et al., 2010; Kuwana et al., 2005). Other studies have suggested that interaction between Bax and BH3 proteins only occurs if both have associated with a membrane (Lovell et al., 2008). This “embedded together” model implies that association with the OMM is a prerequisite for BH3 proteins’ activation of Bax (Leber et al., 2007, 2010). Our study supports the embedded model, in that BH3 proteins do not directly mediate Bax translocation.

The mechanism of Bax retrotranslocation is poorly understood. Recently, Bax was shown to be removed from mitochondria in HCT116 colon carcinoma cells by forming a heterodimer with Bcl-XL, which then dissociated back to the cytosol (Edlich et al., 2011). However, our results suggest that in nontransformed epithelial cells and MEFs, Bcl-XL does not promote Bax retrotranslocation but instead inhibits the mitochondrial-associated form. Bax dissociation was unaffected by ABT-737, and coexpressing Bax and Bcl-XL stabilized the mitochondrial pool of each protein. These data suggest that at least a proportion of membrane-associated Bax is held in a complex with Bcl-XL. This is supported by a recent study suggesting that antiapoptotic Bcl-2 proteins show two distinct modes of action depending on the level of cellular stress (Llambi et al., 2011): one wherein they bind and sequester BH3 moleucles and a second wherein they directly inhibit Bax and Bak. One interpretation of our data, consistent with this model, is that there are two populations of mitochondrial Bax: a dynamic fraction in equilibrium with the cytosol and a more stable fraction bound and inhibited by Bcl-XL. Inhibiting survival signals would increase the mitochondrial fraction requiring inhibition by Bcl-XL.

The possibility that cells contain distinct populations of Bax might also reconcile the conflicting mechanisms proposed for retrotranslocation. Our data suggest that mitochondrial Bax that retrotranslocates has been inserted into the OMM via helix 9. In keeping with this, mitochondrial Bax inhibited by Bcl-XL was membrane inserted (Llambi et al., 2011). However, the reversible association of Bax with liposomes did not involve its membrane insertion (Yethon et al., 2003), and in HCT116 cells, Bax retrotranslocated by Bcl-XL had not been inserted into the OMM (Todt et al., 2013). It is possible that Bax’s association with mitochondria occurs in a stepwise manner. An initial interaction may occur prior to insertion into the membrane, and here Bcl-XL can remove it back to the cytosol. In contrast, Bax that progresses to membrane insertion via helix 9 instead requires Bcl-XL to supress its activation. Cell-type-specific signaling may determine the proportion of Bax at each stage and thus the mechanism seen to remove it. A complex picture of Bax dynamics thus emerges, with multiple steps in its association with and insertion into mitochondria. Importantly, both of these steps are reversible and occur prior to cell commitment to apoptosis.

The question remains of how Bax accumulation on mitochondria is controlled. One possibility is through Bax-TA stability in the OMM. This is suggested by the slower dissociation of BaxS184V, a substitution that increases the hydrophobicity of the TA and thus might stabilize its membrane insertion. Importantly, the Bak TA is significantly more hydrophobic and does not retrotranslocate. The Bax TA appears to constitutively retrotranslocate, which might be controlled by enzymes such as p97VCP, an AAA+ adenosine triphosphatase (ATPase) that can remove membrane-inserted ER (Zhong et al., 2004) and mitochondrial proteins (Tanaka et al., 2010; Xu et al., 2011). Although we found no evidence that p97VCP promoted Bax retrotranslocation (data not shown), regulating stability of membrane insertion requires further investigation. Another possibility is via the Bax C-terminal conformation (Schinzel et al., 2004). Conversion of Bax back to its cytosolic conformation could be catalyzed by a peptidyl-prolyl isomerase, such as Pin1, acting on Pro168 (Suzuki et al., 2000). Pin1 suppresses Bax activation in eosinophils, regulated by Bax phosphorylation downstream of cytokine receptors (Shen et al., 2009).

The exposure of the N terminus of Bax, detected by the monoclonal antibody 6A7, is linked with its activation to induce MOMP. It is not known, however, whether this conformational change occurs concomitant with Bax mitochondrial association or only at the terminal step when MOMP occurs. Bcl-XL can prevent 6A7-epitope exposure of Bax on membranes (Billen et al., 2008). We found that when Bax initially accumulated on mitochondria, it was 6A7 negative and dependent upon Bcl-XL for sequestration. Treatment with ABT-737 resulted in 6A7-epitope exposure of Bax. It is possible that Bax has changed into a 6A7-exposed conformation, but that the epitope is bound and hidden by Bcl-XL. However, the fact that mitochondrial Bax can retrotranslocate to the cytosol when survival signals are restored indicates that it is not yet in a terminally activated conformation.

The conundrum of why Bax and Bak have distinct subcellular distributions in healthy cells is also addressed by our study. We could not measure any significant dissociation of Bak over the same time period in which mitochondrial Bax had completely retrotranslocated to the cytosol. We suggest that Bak provides a baseline of multidomain proapoptotic Bcl-2 protein function on mitochondria, whereas Bax allows cells to adjust their sensitivity to apoptosis by titrating the amount present on the OMM. The amount of Bax required to form a functional pore is around a few hundred molecules per mitochondria (Düssmann et al., 2010). Thus, subtle changes in the mitochondrial concentration of Bax could influence a cell’s sensitivity to undergoing MOMP following BH3 protein activation. Cell sensitivity to MOMP alters according to the milieu of Bcl-2 proteins on the OMM and their relative activation, described as mitochondrial priming (Certo et al., 2006). In cancer cells, this predicts tumor response to chemotherapeutic drugs (Deng et al., 2007; Ni Chonghaile et al., 2011). Cells in the “primed” state are sensitive to ABT-737 and are dependent on antiapoptotic Bcl-2 proteins to suppress Bax and Bak. We now show that the rate of dissociation of Bax from mitochondria also contributes to apoptotic priming.

Many of the interactions between BH3 and multidomain Bcl-2 proteins have been defined using isolated components. However, the regulation of apoptosis in intact cells is subject to rapid and dynamic changes in signal transduction that cannot be replicated in vitro. These signaling pathways are the very things that we now show control rapid changes in Bax distribution between the cytosol and mitochondria. This mechanism may be another way in which aberrant signaling adjusts the sensitivity of cancer cells to apoptosis-inducing chemotherapy.

## Experimental Procedures

### Antibodies and Inhibitors

The antibodies and inhibitors used are as follows: active caspase 3 (R&D Systems); Akt, Akt pT308, and Akt pS473 FAK pY925 (Cell Signaling); GFP, FAK pY397, and FAK pY577 (Invitrogen); FAK (BD Transduction Laboratories); Bax 5B7 and 6A7 (Sigma-Aldrich); ER (Santa Cruz Biotechnology); V5 (Serotec); mtHsp70 (Affinity Bioreagents); secondary antibodies (Jackson Laboratory); ABT-737 (Abbott Laboratories); FAK inhibitor 14 (Tocris Bioscience); SH-6 and etoposide (Calbiochem); and Wortmannin (Cell Signaling).

### Expression Constructs

Constructs expressing GFP-BaxWT, GFP-BaxS184V, GFP-BaxWT/myrAkt, GFP-BaxWT/myrAktER, GFP-BaxWT/myrAktK179M, GFP-BaxWT/myrAktER, and GFP-BaxWT/myrAktK179MER were in pCDH-EF1-MCS-T2A (System Biosciences). GFP-Bax variants were downstream of the T2A sequence, and myrAkt variants were upstream. myrAktER was a gift from Richard Roth (Stanford University). myrAktK179M was generated by QuickChange Site-Directed Mutagenesis (Agilent). mCherry-tBid was a generous gift from David Andrews (McMaster University).

For photoactivatable constructs, GFP was exchanged for paGFP in pCDH vectors (Patterson and Lippincott-Schwartz, 2002), and mRFP-H2B was cloned upstream of the T2A for the identification of transfected cells. pCDNA6-myrFAKWT vectors have been described elsewhere (Zouq et al., 2009). For myrFAKER, the ER sequence (a gift from Martin McMahon, University of California, San Francisco) and cloned in frame with myrFAKWT.

### Cell Culture

FSK-7 MECs (Kittrell et al., 1992) and Bax ^−/−^Bak^−/−^ DKOs were cultured as previously described (Gilmore et al., 2000). Bim^−/−^PUMA^−/−^ DKO MEFs were a generous gift from Emily Cheng (Memorial Sloane-Kettering Cancer Center). Cells were transfected with Lipofectamine Plus (Life Technologies). Stably expressing MECs were generated by lentiviral infection. HEK293T cells were cotransfected with pPsPax2, pMD2G, and the relevant pCDH plasmid, and virus production was induced with 10 mM sodium butyrate. Virus was collected 24 hr later, sterile filtered, and added to MECs in 10 μg/ml Polybrene (Millipore). Cells were passaged over 2 weeks and selected by fluorescence-activated cell sorting (FACS).

### Immunofluorescence Microscopy and Live-Cell Imaging

Cells were immunostained as previously described (Valentijn et al., 2003). For quantitation of Bax localization and apoptosis, images were taken with a 63× (NA 1.4) Plan Apochromat (Plan Apo) objective on a Zeiss Axio Imager M2 using Volocity 5.5.1 (PerkinElmer). Experiments were performed three times and analyzed by ANOVA with Bonferroni’s post hoc test using Prism (GraphPad). Images were taken with 60× (NA 1.4) Plan Apo or 100× (NA 0.5-1.35) Uplan Apo objectives on an Olympus IX71 microscope equipped with a DeltaVision imaging system. Images were deconvolved using softWoRx v.3.0 (Applied Precision).

Live imaging was done on a Zeiss Axio Observer Z1 with a CSU-X1 spinning disk (Yokogawa), using a 63×/1.40 Plan Apo lens, an Evolve EMCCD camera (Photometrics), and a motorized XYZ stage (Applied Scientific Instrumentation) driven by Marianas hardware and SlideBook 5.0 software (Intelligent Imaging Innovations). For FLIP, two regions overlapping the cytosol and nucleus were bleached (two iterations, 488 nm, 100%, 50 ms), and images were captured every 30 s. Images were analyzed using ImageJ. In brief, the background was subtracted, cytosolic content exclusive of the bleached ROIs was selected, and the signal decay was quantified. Data were normalized to 100% fluorescence post bleaching. Statistical analysis was carried out by ANOVA with Bonferroni’s post hoc test (Prism, GraphPad). For paGFP, one ROI was photoactivated (405 nm laser, 100%, 50 ms), and images were captured over 10 min at 30 s intervals. The mitochondrial association rate was determined in a manner similar to that used for FLIP experiments.

## Supplemental Information

Document S1. Figures S1–S5 and Supplemental Experimental Procedures

Movie S1. FLIP Analysis of GFP-Bax and GFP-BaxS184V in MECs, Related to Figure 1 Movie S1 is related to Figure 1A and shows dissociation of mitochondrial Bax after photobleaching in MECs stably expressing (left to right) GFP-Bax, GFP-BaxS184V, GFP-BaxS184V in fixed cells, and GFP-Bax in cells treated with ABT-737.

Movie S2. FLIP Analysis of GFP-Bax and GFP-BaxS184V in *Bax*^−*/*−^*Bak*^−*/*−^ DKO MEFs, Related to Figure 1 Movie S2 is related to Figure 1C and shows (left to right) *Bax*^−*/*−^*Bak*^−*/*−^ DKO MEFs transfected with GFP-Bax, GFP-Bax treated with ABT-737, and GFP-BaxS184V.

Movie S3. FLIP Analysis of GFP-Bax in *Bax*^−*/*−^*Bak*^−*/*−^ and *Bim*^−*/*−^*PUMA*^−*/*−^ DKO MEFs, Related to Figure 1 Movie S3 relates to Figure 1D and shows (left to right) *Bax*^−*/*−^*Bak*^−*/*−^ DKO MEFs expressing GFP-Bax, *Bim*^−*/*−^*PUMA*^−*/*−^ DKO MEFs expressing GFP-Bax, and *Bax*^−*/*−^*Bak*^−*/*−^ DKO MEFs coexpressing GFP-Bax and mCherry-tBid (only the GFP-Bax is shown in the movie; see Figure 1D for the corresponding mCherry-tBid images).

Movie S4. Photoactivation of paGFP-Bax Expressed in MECs, Related to Figure 2 Movie S4 relates to Figure 2A, and shows the accumulation of paGFP-BaxS184V on mitochondria. The cell also shows the coexpressed mRFP-H2B.

Movie S5. Photoactivation of paGFP-Bax in MECs Followed by FLIP, Related to Figure 2 Movie S5 relates to Figure 2B. MECs were transfected with paGFP-Bax, which was first photoactivated and then photobleached for FLIP.

Movie S6. FRAP Analysis of GFP-BclXL and DsRed-mito in MECs, Related to Figure 3 Movie S6 relates to Figure 3B and shows FRAP on MECs expressing both GFP-BclXL (left panel) and DsRed-mito (right panel).

Movie S7. FRAP Analysis of mRFP-Bcl-XL Expressed in *Bax*^−*/*−^*Bak*^−*/*−^ DKO MEFs, Related to Figure 3 Movie S7 relates to Figure 3C and shows FRAP on DKO MEFs expressing mRFP-BclXL alone (left panel) or with GFP-Bax (right panel; only the mRFP-BclXL is shown in the movie; see Figure 3C for the corresponding GFP-Bax images).

Movie S8. FLIP Analysis Showing the Effect of FAK Inhibition on GFP-Bax Dissociation, Related to Figure 6 Movie S8 is related to Figure 6C and display the influence of FAK inhibition on the dissociation of stably expressed GFP-Bax in MECs. The left panel shows untreated cells; the right panel shows cells treated with FAK inh.14.

Movie S9. FLIP Analysis of GFP-Bax in MECs Coexpressing Active or Kinase-Dead Akt, Related to Figure 6 Movie S9 is related to Figure 6F and demonstrates that changes in Akt signaling lead to a shift of the Bax equilibrium in MECs. The left panel shows GFP-Bax alone, the middle panel shows GFP-Bax coexpressed with myrAktK179M, and the right panel shows GFP-Bax coexpressed with myrAkt.

## References

[bib1] Billen L.P., Kokoski C.L., Lovell J.F., Leber B., Andrews D.W. (2008). Bcl-XL inhibits membrane permeabilization by competing with Bax. PLoS Biol..

[bib2] Borgese N., Brambillasca S., Colombo S. (2007). How tails guide tail-anchored proteins to their destinations. Curr. Opin. Cell Biol..

[bib3] Bouillet P., Strasser A. (2002). BH3-only proteins - evolutionarily conserved proapoptotic Bcl-2 family members essential for initiating programmed cell death. J. Cell Sci..

[bib4] Certo M., Del Gaizo Moore V., Nishino M., Wei G., Korsmeyer S., Armstrong S.A., Letai A. (2006). Mitochondria primed by death signals determine cellular addiction to antiapoptotic BCL-2 family members. Cancer Cell.

[bib5] Chipuk J.E., Moldoveanu T., Llambi F., Parsons M.J., Green D.R. (2010). The BCL-2 family reunion. Mol. Cell.

[bib6] Deng J., Carlson N., Takeyama K., Dal Cin P., Shipp M., Letai A. (2007). BH3 profiling identifies three distinct classes of apoptotic blocks to predict response to ABT-737 and conventional chemotherapeutic agents. Cancer Cell.

[bib7] Düssmann H., Rehm M., Concannon C.G., Anguissola S., Würstle M., Kacmar S., Völler P., Huber H.J., Prehn J.H. (2010). Single-cell quantification of Bax activation and mathematical modelling suggest pore formation on minimal mitochondrial Bax accumulation. Cell Death Differ..

[bib8] Edlich F., Banerjee S., Suzuki M., Cleland M.M., Arnoult D., Wang C., Neutzner A., Tjandra N., Youle R.J. (2011). Bcl-x(L) retrotranslocates Bax from the mitochondria into the cytosol. Cell.

[bib9] Gavathiotis E., Reyna D.E., Davis M.L., Bird G.H., Walensky L.D. (2010). BH3-triggered structural reorganization drives the activation of proapoptotic BAX. Mol. Cell.

[bib10] Gilmore A.P., Metcalfe A.D., Romer L.H., Streuli C.H. (2000). Integrin-mediated survival signals regulate the apoptotic function of Bax through its conformation and subcellular localization. J. Cell Biol..

[bib11] Hsu Y.T., Youle R.J. (1998). Bax in murine thymus is a soluble monomeric protein that displays differential detergent-induced conformations. J. Biol. Chem..

[bib12] Kittrell F.S., Oborn C.J., Medina D. (1992). Development of mammary preneoplasias in vivo from mouse mammary epithelial cell lines in vitro. Cancer Res..

[bib13] Kohn A.D., Barthel A., Kovacina K.S., Boge A., Wallach B., Summers S.A., Birnbaum M.J., Scott P.H., Lawrence J.C., Roth R.A. (1998). Construction and characterization of a conditionally active version of the serine/threonine kinase Akt. J. Biol. Chem..

[bib14] Kuwana T., Bouchier-Hayes L., Chipuk J.E., Bonzon C., Sullivan B.A., Green D.R., Newmeyer D.D. (2005). BH3 domains of BH3-only proteins differentially regulate Bax-mediated mitochondrial membrane permeabilization both directly and indirectly. Mol. Cell.

[bib15] Leber B., Lin J., Andrews D.W. (2007). Embedded together: the life and death consequences of interaction of the Bcl-2 family with membranes. Apoptosis.

[bib16] Leber B., Lin J., Andrews D.W. (2010). Still embedded together binding to membranes regulates Bcl-2 protein interactions. Oncogene.

[bib17] Lindsten T., Ross A.J., King A., Zong W.X., Rathmell J.C., Shiels H.A., Ulrich E., Waymire K.G., Mahar P., Frauwirth K. (2000). The combined functions of proapoptotic Bcl-2 family members bak and bax are essential for normal development of multiple tissues. Mol. Cell.

[bib18] Llambi F., Moldoveanu T., Tait S.W., Bouchier-Hayes L., Temirov J., McCormick L.L., Dillon C.P., Green D.R. (2011). A unified model of mammalian BCL-2 protein family interactions at the mitochondria. Mol. Cell.

[bib19] Lovell J.F., Billen L.P., Bindner S., Shamas-Din A., Fradin C., Leber B., Andrews D.W. (2008). Membrane binding by tBid initiates an ordered series of events culminating in membrane permeabilization by Bax. Cell.

[bib20] Nechushtan A., Smith C.L., Hsu Y.T., Youle R.J. (1999). Conformation of the Bax C-terminus regulates subcellular location and cell death. EMBO J..

[bib21] Ni Chonghaile T., Sarosiek K.A., Vo T.T., Ryan J.A., Tammareddi A., Moore Vdel.G., Deng J., Anderson K.C., Richardson P., Tai Y.T. (2011). Pretreatment mitochondrial priming correlates with clinical response to cytotoxic chemotherapy. Science.

[bib22] Oltersdorf T., Elmore S.W., Shoemaker A.R., Armstrong R.C., Augeri D.J., Belli B.A., Bruncko M., Deckwerth T.L., Dinges J., Hajduk P.J. (2005). An inhibitor of Bcl-2 family proteins induces regression of solid tumours. Nature.

[bib23] Patterson G.H., Lippincott-Schwartz J. (2002). A photoactivatable GFP for selective photolabeling of proteins and cells. Science.

[bib24] Schaller M.D. (2010). Cellular functions of FAK kinases: insight into molecular mechanisms and novel functions. J. Cell Sci..

[bib25] Schinzel A., Kaufmann T., Schuler M., Martinalbo J., Grubb D., Borner C. (2004). Conformational control of Bax localization and apoptotic activity by Pro168. J. Cell Biol..

[bib26] Shen Z.J., Esnault S., Schinzel A., Borner C., Malter J.S. (2009). The peptidyl-prolyl isomerase Pin1 facilitates cytokine-induced survival of eosinophils by suppressing Bax activation. Nat. Immunol..

[bib27] Suzuki M., Youle R.J., Tjandra N. (2000). Structure of Bax: coregulation of dimer formation and intracellular localization. Cell.

[bib28] Tanaka A., Cleland M.M., Xu S., Narendra D.P., Suen D.F., Karbowski M., Youle R.J. (2010). Proteasome and p97 mediate mitophagy and degradation of mitofusins induced by Parkin. J. Cell Biol..

[bib29] Todt F., Cakir Z., Reichenbach F., Youle R.J., Edlich F. (2013). The C-terminal helix of Bcl-x(L) mediates Bax retrotranslocation from the mitochondria. Cell Death Differ..

[bib30] Valentijn A.J., Metcalfe A.D., Kott J., Streuli C.H., Gilmore A.P. (2003). Spatial and temporal changes in Bax subcellular localization during anoikis. J. Cell Biol..

[bib31] Valentijn A.J., Upton J.P., Bates N., Gilmore A.P. (2008). Bax targeting to mitochondria occurs via both tail anchor-dependent and -independent mechanisms. Cell Death Differ..

[bib32] Valentijn A.J., Upton J.P., Gilmore A.P. (2008). Analysis of endogenous Bax complexes during apoptosis using blue native PAGE: implications for Bax activation and oligomerization. Biochem. J..

[bib33] Walensky L.D., Gavathiotis E. (2011). BAX unleashed: the biochemical transformation of an inactive cytosolic monomer into a toxic mitochondrial pore. Trends Biochem. Sci..

[bib34] Wei M.C., Zong W.X., Cheng E.H., Lindsten T., Panoutsakopoulou V., Ross A.J., Roth K.A., MacGregor G.R., Thompson C.B., Korsmeyer S.J. (2001). Proapoptotic BAX and BAK: a requisite gateway to mitochondrial dysfunction and death. Science.

[bib35] Xu S., Peng G., Wang Y., Fang S., Karbowski M. (2011). The AAA-ATPase p97 is essential for outer mitochondrial membrane protein turnover. Mol. Biol. Cell.

[bib36] Yethon J.A., Epand R.F., Leber B., Epand R.M., Andrews D.W. (2003). Interaction with a membrane surface triggers a reversible conformational change in Bax normally associated with induction of apoptosis. J. Biol. Chem..

[bib37] Youle R.J., Strasser A. (2008). The BCL-2 protein family: opposing activities that mediate cell death. Nat. Rev. Mol. Cell Biol..

[bib38] Zhong X., Shen Y., Ballar P., Apostolou A., Agami R., Fang S. (2004). AAA ATPase p97/valosin-containing protein interacts with gp78, a ubiquitin ligase for endoplasmic reticulum-associated degradation. J. Biol. Chem..

[bib39] Zouq N.K., Keeble J.A., Lindsay J., Valentijn A.J., Zhang L., Mills D., Turner C.E., Streuli C.H., Gilmore A.P. (2009). FAK engages multiple pathways to maintain survival of fibroblasts and epithelia: differential roles for paxillin and p130Cas. J. Cell Sci..

