# Supplementary material for: Bax Exists in a Dynamic Equilibrium between the Cytosol and Mitochondria to Control Apoptotic Priming
Source: Mol Cell. 2013 Mar 7;49(5):959–71. doi: 10.1016/j.molcel.2012.12.022 (PMC3594749; doi:10.1016/j.molcel.2012.12.022)
Supplement: Document S1. Figures S1–S5 and Supplemental Experimental Procedures [file mmc1.pdf]

Molecular Cell, Volume *49*

Supplemental Information

Bax Exists in a Dynamic Equilibrium between the Cytosol and Mitochondria to Control Apoptotic Priming

Barbara Schellenberg, Pengbo Wang, James A. Keeble, Ricardo Rodriguez-Enriquez , Scott Walker, Thomas Owens, Fiona Foster, Jolanta Tanianis-Hughes, Keith Brennan, Charles H. Streuli and Andrew P. Gilmore.

Supplemental Information Index:

1. Supplemental figures.

2. List of supplemental movie files.

3. Supplemental Experimental Procedures.

4. Supplemental References.

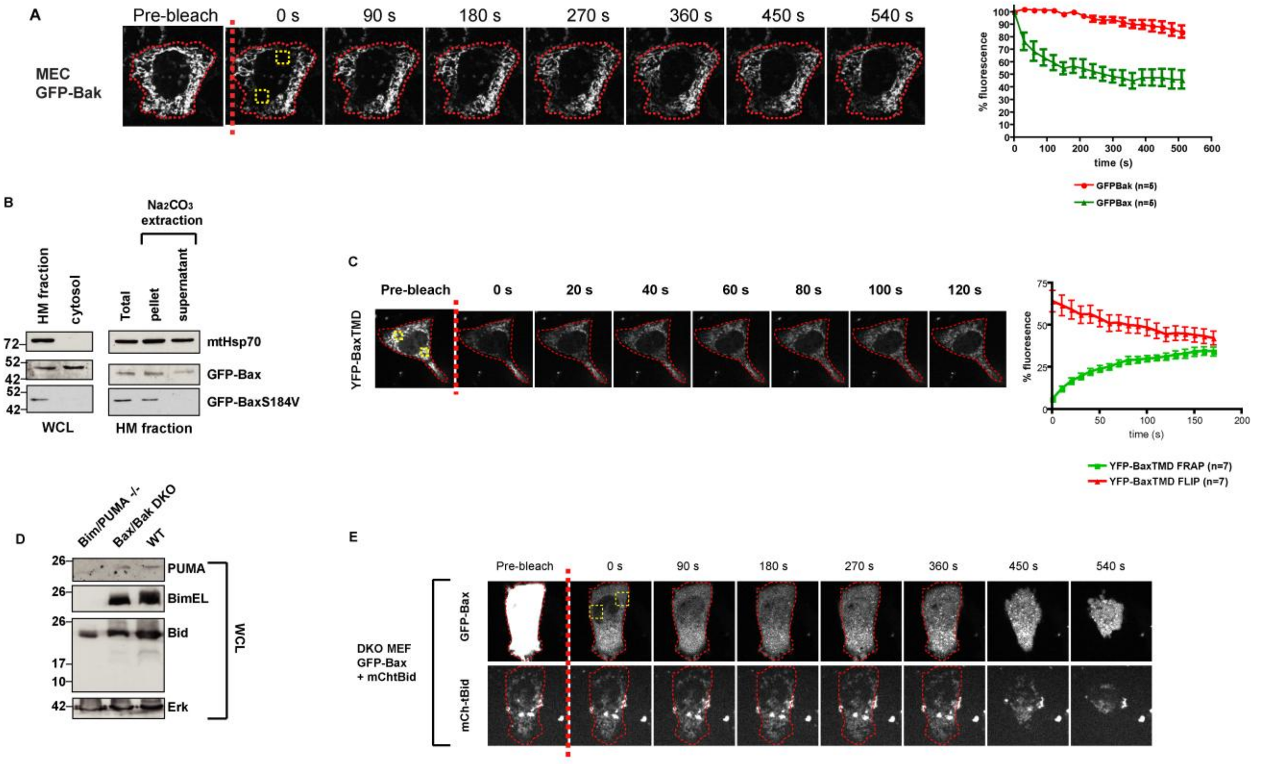

Schellenberg et al. Suppl. Figure 1

Figure S1. **Bax Constantly Dissociates from Mitochondria in Nonapoptotic Cells, Related to Figure 1**

A. FLIP analysis of GFP-Bak transiently expressed in FSK-7 cells. GFP-Bak was photobleached by two 50 ms iterations at 488 nm in the ROI marked by the yellow squares. The dissociation of the mitochondrial GFP signal was monitored as previously described for GFP-Bax. The signals obtained post bleaching were analysed as before, and normalised to 100% fluorescence. The green line shows FLIP analysis of GFP-Bax for comparison. Error bars = SEM.

B. Mitochondrial membrane integration of GFP-Bax and GFP-BaxS184V. Cells expressing either GFP-Bax or GFP-BaxS184V were separated into cytosolic or heavy membrane fractions as described in the extended methods. The membrane fraction containing mitochondria was

extracted in sodium carbonate and the insoluble and soluble fractions separated by centrifugation. The fractions were analysed by immunoblotting for GFP and mtHsp-70. WCL = the cytosolic and HM fractions. HM fraction = total HM fraction, pellet (sodium carbonate resistant fraction), supernatant (sodium carbonate sensitive fraction).

C. The Bax tail anchor alone shows rapid association and dissociation from mitochondria. DKO MEFs were transiently transfected with pEYFP-BaxTMD, consisting of the C-terminal 23aa of Bax only, which has previously been shown to directly insert into the outer mitochondrial membrane. The indicated ROIs (yellow) were photobleached as described in the methods. FLIP and analysis was carried out on the unbleached mitochondria, and FRAP was measured within the bleached ROIs. The graph shows association and dissociation from this analysis. Error bars = SEM.

D. Whole cell lysates of wildtype (WT), *Bim*$^{-/-}$*PUMA*$^{-/-}$, and *Bax*$^{-/-}$*Bak*$^{-/-}$MEFs were immunoblotted for PUMA, Bim and Bid. Erk indicates equivalent protein loading.

E. FLIP analysis of GFP-Bax expressed in either *Bax*$^{-/-}$*Bak*$^{-/-}$ DKO MEFs co-expressing mCherry-tBid. This is similar to that shown in Fig. 1D, but shows an example of the proportion of cells that underwent apoptosis during the course of filming.

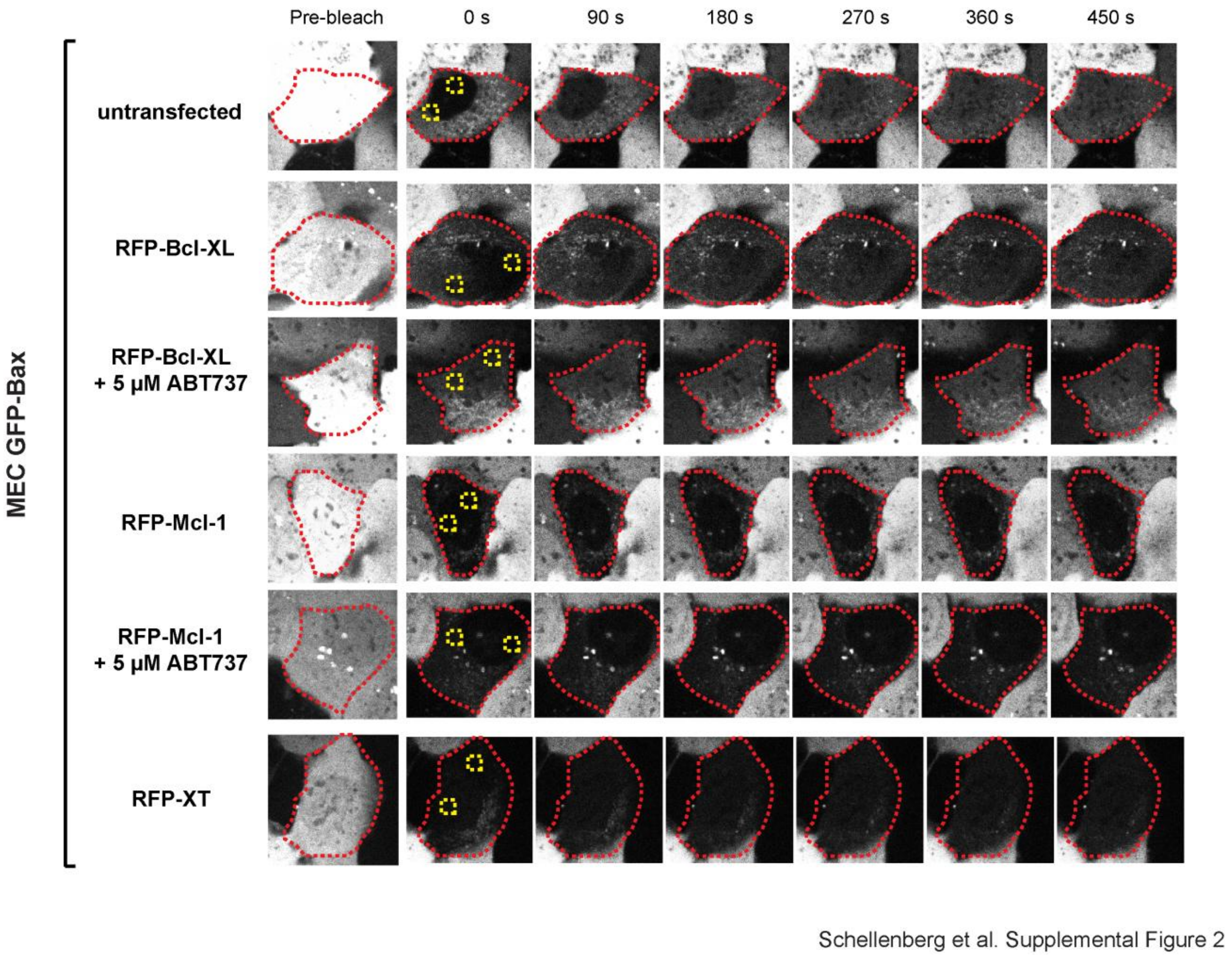

Figure S2. FLIP Analysis of GFP-Bax in Cells Coexpressing Bcl-XL and Mcl-1, Related to Figure 3

Representative movie sequences of MECs stably expressing GFP-Bax and analysed in Fig. 1D. MECs stably expressing GFP-Bax were transfected with expression vectors for either mRFP-Bcl-XL or mRFP-Mcl-1. 18 hours post transfection, FLIP analysis was carried out as in Fig. 1A in the presence or absence of ABT-737.

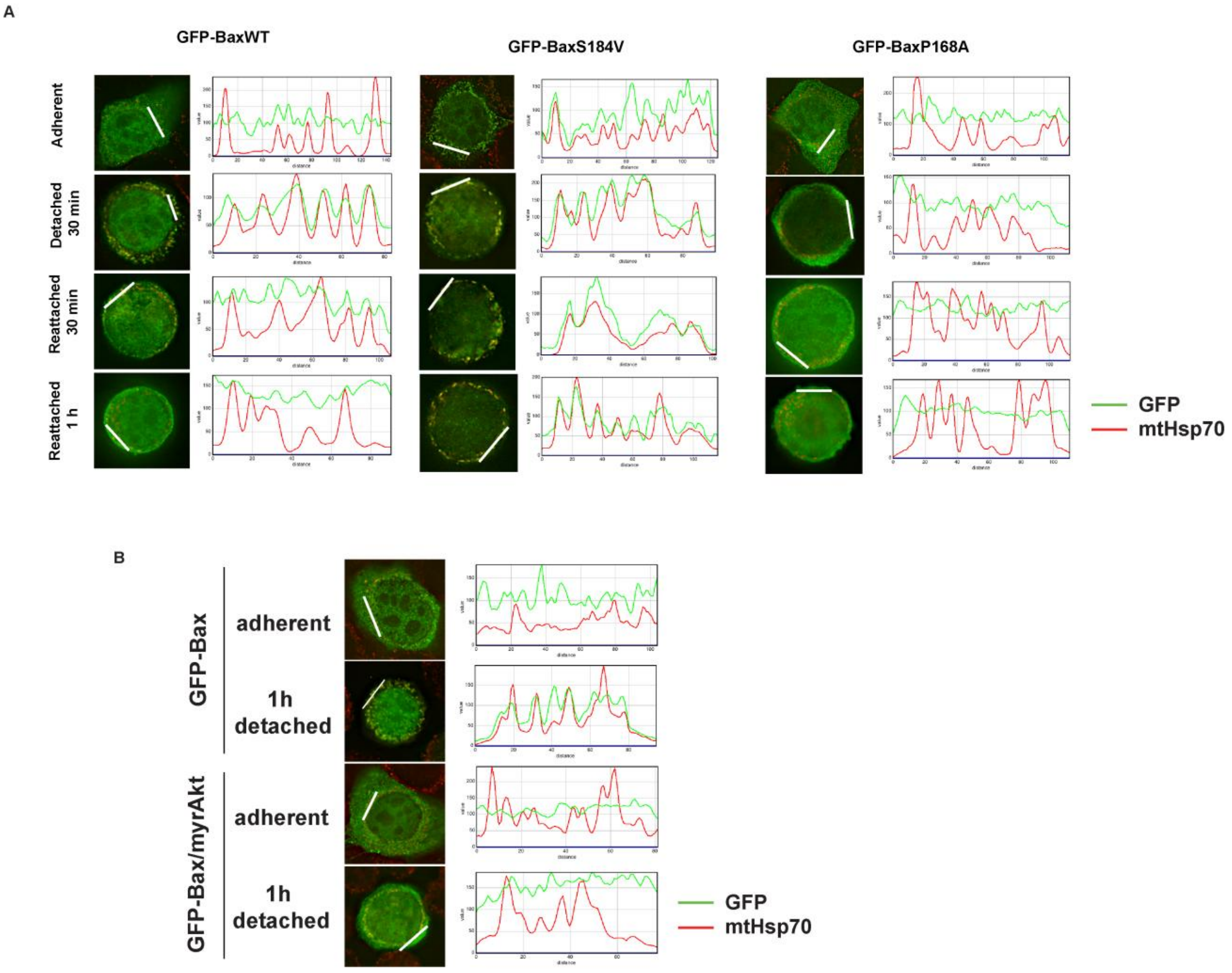

Schellenberg et al. Suppl. Figure 3

Figure S3. Colocalization Analysis of GFP-Bax and Mitochondria, Related to Figures 4A and 4D

A. Colocalisation analysis of GFP-Bax (see Fig. 3A), GFP-BaxS184V, and a translocation incompetent variant, GFP-BaxP168A, with mitochondria. MEC expressing each of the three GFP-Bax variants were left adherent, detached for 30 minutes, or detached and allowed to reattach to ECM for the indicated times. Cells were fixed and stained as described in the extended methods, and images captured using a DeltaVision system. Deconvolved images were analysed in ImageJ using RGB line profiles to examine co-localisation between GFP and mtHsp-70. Line profiles are shown to the right of each image, with GFP=green and mtHsp70= red.

B. Adherent and detached (1 hour) MEC expressing GFP-Bax alone or along with myrAkt were analysed as in A, above.

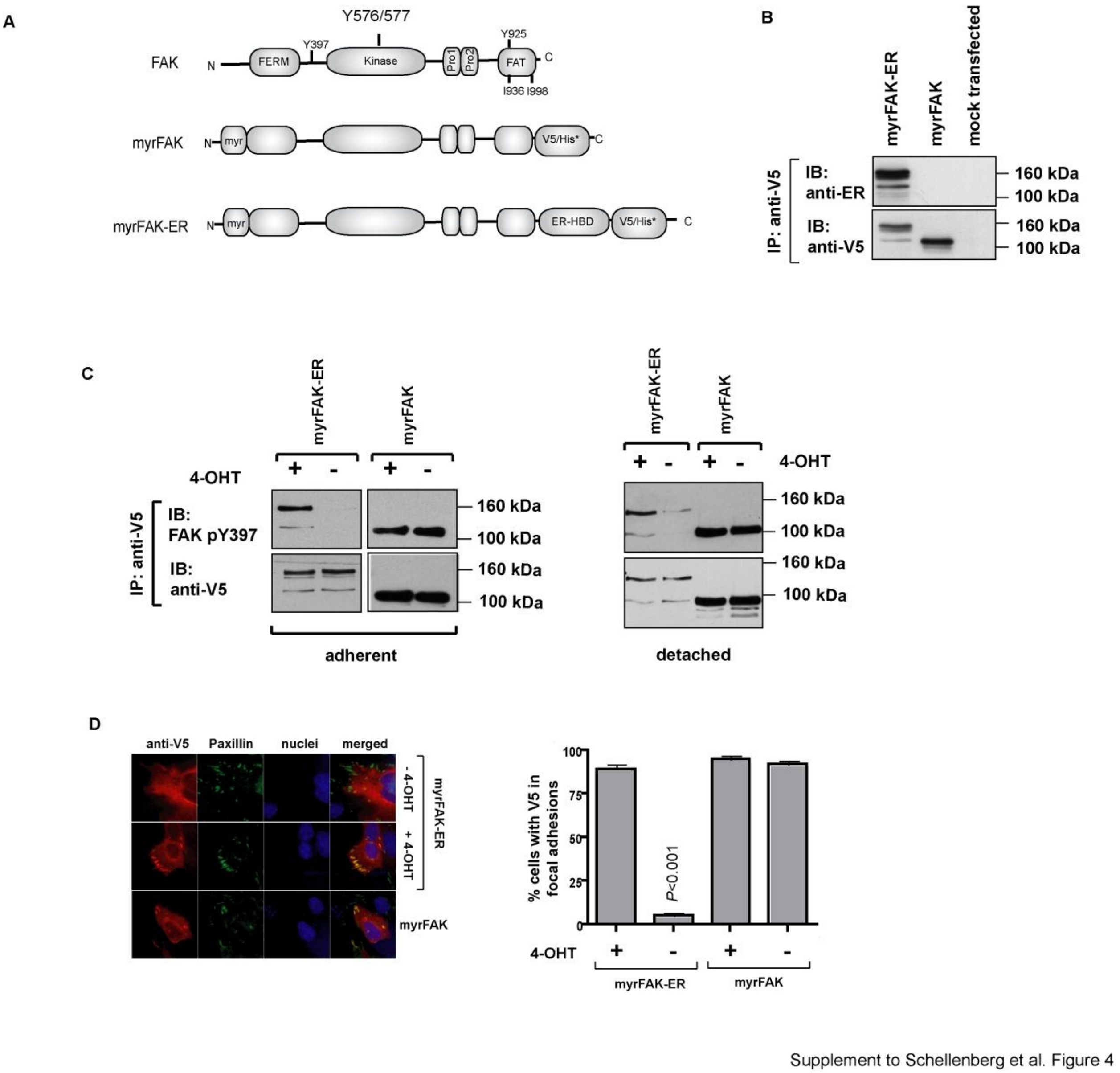

Figure S4. Generation and Characterization of Conditionally Active FAK, Related to Figure 5

A. Schematic representation of the construction of myrFAK and myrFAKER.

B. Expression of myrFAK and myrFAKER in FSK-7 cells. myrFAK and myrFAKER were immunoprecipitated with anti-V5 and detected by immunoblotting with anti-V5 and anti-ER.

C. Activation of myrFAKER is dependent upon 4-hydroxytamoxifen in both adherent and detached FSK-7 cells. Adherent or detached MECs expressing myrFAK or myrFAKER were left

untreated or treated with 4-OHT for 1 hour. myrFAK and myrFAKER were immunoprecipitated with anti-V5 and detected by immunoblotting with anti-phospho-FAK Y397 and anti-V5.

D. myrFAKER recruitment to focal adhesions in adherent cells dependent upon 4-OHT. Mouse embryonic fibroblasts (MEFs) transiently expressing myrFAK or myrFAKER were treated as indicated, before fixing and immunostaining with anti-V5 and anti-paxillin to indicate focal adhesions. The number of cells with V5 staining in focal adhesions was quantified. Error bars = SEM.

A

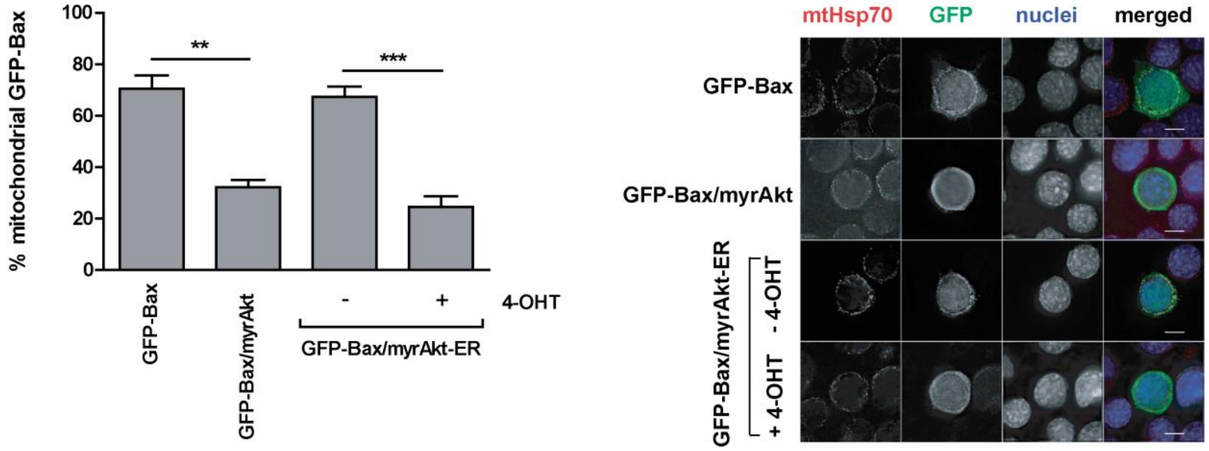

B

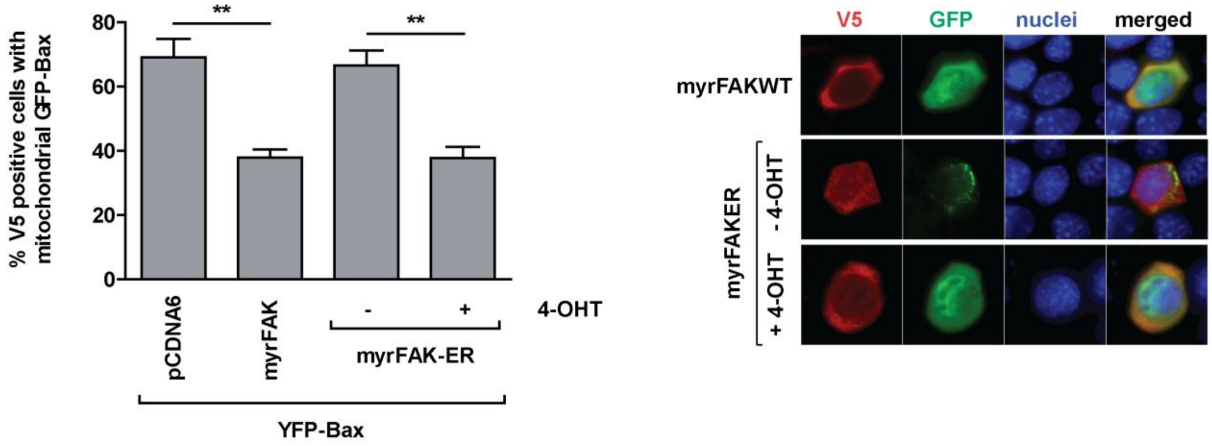

C

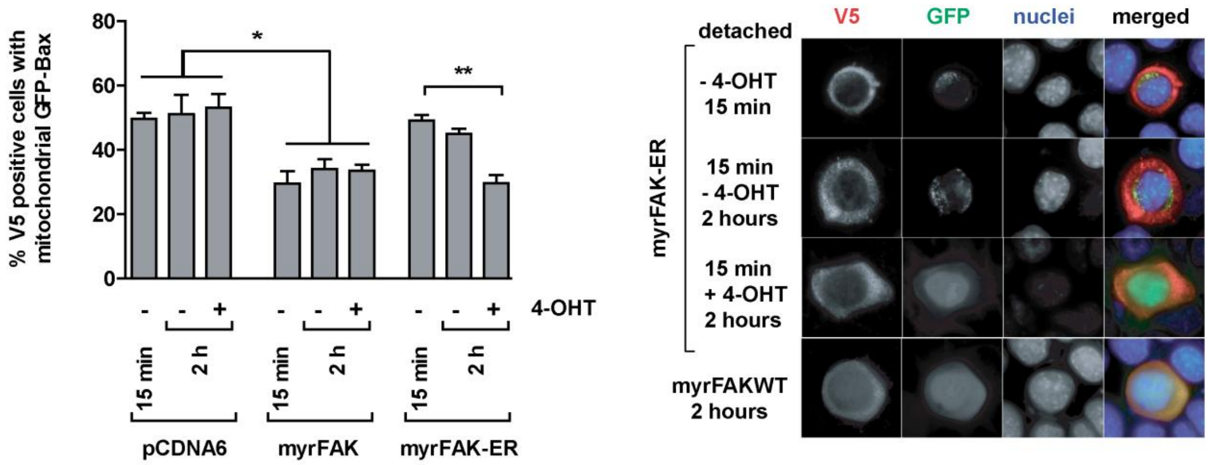

Schellenberg et al. Supplemental Figure 5

Figure S5. myrAktER and myrFAK ER Prevent GFP-Bax Accumulation on Mitochondria, Related to Figure 5

A. MECs transiently co-expressing GFP-Bax, myrAkt or myrAktER, as indicated, were detached from ECM for 30 minutes. Indicated cells had been pre-treated for 1 hour with 4-hydroxytamoxifen. Cells were immunostained with anti-GFP and anti-mtHsp70, and nuclei stained with Hoechst. The number of cells with predominantly mitochondrial GFP-Bax was quantified.

B. MECs transiently co-expressing GFP-Bax, myrFAK or myrFAKER, as indicated, were detached from ECM for 30 minutes. Indicated cells had been pre-treated for 1 hour with 4-hydroxytamoxifen. Cells were immunostained with anti-GFP and anti-mtHsp70, and nuclei stained with Hoechst. The number of cells with predominantly mitochondrial GFP-Bax was quantified.

C. Activation of myrFAKER leads to dissociation of mitochondrial Bax. MEC expressing either GFP-Bax alone, or with myrFAK or myrFAKER, were detached for 15 min. Cells were then left or treated with 4-OHT for 2 hours. Cells were immunostained for anti-GFP and anti-V5 and mitochondrial localisation of GFP-Bax quantified in the V5-postive cells.

In all cases, data represent the mean of three independent experiments. Error bars = SEM. Data was analysed by ANOVA, * = $p<0.05$; ** = $p<0.001$.

Legends for Supplemental Movies

Movies S1– S3 relate to Figure 1.

Movie S1 is related to Fig. 1A and shows dissociation of mitochondrial Bax after photobleaching in MECs stably expressing (left to right) GFP-Bax, GFP-BaxS184V, GFP-BaxS184V in fixed cells, and GFP-Bax in cells treated with ABT737.

Movies S2 is related to Fig. 1C, and shows (left to right) Bax/Bak DKO MEFs transfected with GFP-Bax, GFP-Bax treated with ABT737, and GFP-BaxS184V.

Movies S3 relates to Fig. 1D, and shows (left to right) Bax/Bak DKO MEFs expressing GFP-Bax, Bim/PUMA DKO MEFs expressing GFP-Bax, and Bax/Bak DKO-MEFs co-expressing GFP-Bax and mCherry-tBid (only the GFP-Bax is shown in the movie, see Fig. 1D for the corresponding mCherry-tBid images).

Movies S4 and S5 relate to Figure 2.

Movie S4 relates to Fig. 2A, and shows accumulation of paGFP-BaxS184V on mitochondria. The cell also shows the co-expressed mRFP-H2B.

Movie S5 relates to Fig. 2B. MECs were transfected with photoactivatable paGFP-Bax, which was first photo-activated and then photobleached for FLIP.

Movies S6 and S7 relate to Figure 3.

Movies S6 relates to Fig. 3B, and show FRAP on MEC expressing both GFP-BclXL (left panel) and DsRed-mito (right panel).

Movies S7 relates to Fig. 3C, and show FRAP on DKO MEFs expressing mRFP-BclXL alone (left panel) or with GFP-Bax (right panel, only the mRFP-BclXL is shown in the movie, see Fig. 3C for the corresponding GFP-Bax images).

Movies S8 and S9 relate to Figure 6.

Movie S8 is related to Fig. 6C and display the influence of FAK inhibition on the dissociation of stably expressed GFP-Bax in MECs. The left panel shows untreated cells, the right panel cells treated with FAK inh.14.

Movies S9 is related to Fig. 6F and demonstrates that changes in Akt signalling lead to a shift of the Bax equilibrium in MECs. The left panel shows GFP-Bax alone, the middle panel shows GFP-Bax co-expressed with myrAktK179M, and the right panel GFP-Bax co-expressed with myrAkt.

Supplemental Experimental Procedures

Antibodies and inhibitors.

Antibodies used were from the following suppliers: Active caspase 3 (R&D Systems); Akt, Akt pT308, Akt pS473 FAK pY925 (all from Cell Signalling); GFP, FAK pY397 and FAK pY577 (all from Invitrogen); FAK (BD Transduction Lab); Bax 5B7 and 6A7 (Sigma); oestrogen receptor α (Santa Cruz); anti-V5 (Serotec); mtHsp70 (Affinity Bioreagents); all secondary antibodies, Alexa 488, Alexa 594 and HRP conjugates (Jackson Labs).

Small MW inhibitors used were obtained from the following: ABT-737 (Abbott); FAK inhibitor 14 (Tocris Bioscience); SH-6 and Etoposide (Calbiochem); Wortmannin (Cell Signalling).

Cell culture, transfection and lentiviral infection.

FSK-7 MECs (Kittrell et al., 1992) were cultured as previously described (Gilmore et al., 2000) in DMEM/F12 supplemented with 2% fetal calf serum, 5 ng/ml epidermal and growth factor and 880 nM insulin. *Bax*$^{-/-}$*Bak*$^{-/-}$ DKO MEFs and *Bim*$^{-/-}$*PUMA*$^{-/-}$ DKO MEFs (a generous gift from Dr. Emily Cheng, Memorial Sloane-Kettering Cancer Center, New York) were cultured in DMEM supplemented with 10% fetal calf serum and 50 µM β-mercaptoethanol. For anoikis assays, FSK-7 cells were trypsinised and resuspended in complete growth media before plating onto poly-HEMA coated culture dishes, as previously described (Gilmore et al., 2000). Cells were harvested by centrifugation at 5000 x g at appropriate times, and processed either for Western blotting or immunofluorescecne, as described below.

For transient expression, FSK-7 or MEFs were transfected with 1 µg of plasmid DNA using Lipofectamine Plus (Life Technologies). Cells were used 18 h post transfection.

For stable expression, FSK-7 cells were infected using lentiviruses. HEK 293T cells were co-transfected with pPsPax2, pMD2G, and the relevant pCDH plasmids (see below), and virus production induced with 10 mM sodium butyrate. Virus was collected 24 h later, sterile filtered

and added to MECs in 10 μg/ml Polybrene (Millipore). Cells were passaged for two weeks and selected by FACS, based upon GFP-Bax expression, using a FACS Aria (BD Biosciences).

Plasmid and lentiviral expression constructs.

Two different strategies were used for co-expression of either Akt or FAK along with fluorescently tagged Bax.

For expression of active FAK (myrFAK), we co-transfected cells with two plasmids, pEGFP-Bax and pCDNA6-myrFAK. pCDNA6-myrFAK plasmids have previously been described (Zouq et al., 2009), and the expressed myrFAK has a C-terminal V5 epitope tag to identify expressing cells. To generate myrFAKER, a modified ERα sequence (Littlewood et al., 1995) (a gift from Martin McMahon, UCSF) was amplified by PCR and cloned in frame between myrFAKWT and the coding sequence for the V5 epitope. Co-expressing cells were identified by immunostaining with anti-GFP and anti-V5.

For Akt and Bax expression, we used a single lentiviral vector expressing both genes from an EF1α promoter, linked by a self-cleaving 2A peptide, negating the requirement for directly identifying both expressed proteins in transfected cells. Using the pCDH-EF1a-T2A vector (System Biosciences), we sub-coned GFP-Bax and its variants downstream of the T2A sequence, and the myrAkt and variants upstream. This was used for the following combinations; pCDH-GFP-BaxWT, pCDH-GFP-BaxS184V, pCDH-GFP-BaxWT/myrAkt, pCDH-GFP-BaxWT/myrAktER, pCDH-GFP-BaxWT/myrAktK179M, pCDH-GFP-BaxWT/myrAktER and pCDH-GFP-BaxWT/myrAktK179MER. GFP-Bax variants were downstream of the T2A sequence, and myrAkt upstream. myrAktER was a gift from Richard Roth (Stanford, USA). myrAktK179M was generated by Quick Change Site-Directed Mutagenesis (Agilent).

For photoactivatable GFP-Bax, we exchanged EGFP coding sequence with that for paGFP (Patterson and Lippincott-Schwartz, 2002) in the pCDH-Bax and pCDH-BaxS184V vectors. To allow identification of expressing cells prior to photoactivation, mRFP-H2B was sub-cloned upstream of the T2A sequence.

Other Bcl-2 family proteins were expressed as follows; GFP-Bcl-XL and GFP-Bak were expressed in pEGFP-C1 (Clontech); mRFP-Mcl-1 and mRFP-Bcl-XL were made by swapping

the EYFP coding sequence in pEYFP-C1 (Clontech) with the coding sequence for mRFP (Campbell et al., 2002). The coding sequences for Mcl-1 and Bcl-XL were amplified by PCR and subcloned in frame with the mRFP coding sequence. The vector encoding mCherry-tBid was a generous gift from David Andrews (McMaster University, Canada). pEYPF-BaxTMD has been previously described (Valentijn et al., 2008), and consists of coding sequence for the C-terminal 23 amino acids of murine Bax. mRFP-XT consists of the C-terminal tail anchor from human Bcl-XL fused to mRFP. It was generated from pEYFP-XT, which has been previously described (Valentijn et al., 2003), and consists of the C-terminal 25 amino acids of Bcl-XL. The YFP-coding sequence was removed and replaced with that of mRFP.

pDsRed-mito was obtained from (Clontech), and contains DsRed fused to the mitochondrial import sequence from cytochrome c oxidase subunit VIII.

Immunofluorescence microscopy.

Cells were immunostained as previously described (Valentijn et al., 2003). Breifly, detached cells were cytospun onto polysine slides (Merck) using a cytological centrifuge (Shandon) after the required time in suspension on poly-HEMA coated dishes. Adherent cells were cultured on glass coverslips. Cells were fixed in 4% formaldehyde in PBS, and immunostained. myrFAK and GFP-Bax expressing cells were immunostained with anti-V5 and anti-GFP to identify co-expressing cells. pCDH based experiments were immunostained with anti-mtHsp70 and anti-GFP. For quantitation of Bax localisation, immunostained cells were imaged with a 63x (NA 1.4) Plan Apochromat objective on a Zeiss Axio Imager M2 using Volocity 5.5.1 (PerkinElmer). For quantification, between 100 and 200 cells were imaged for each condition per experiment. Experiments were performed independently three times and analysed by ANOVA with Bonferroni's post-test using Prism (GraphPad). For figures and line plots, images were taken with 60x (NA 1.4) Plan-Apo or 100x (NA 0.5-1.35) Uplan Apo objectives, on an Olympus IX71 microscope equipped with Delta Vision imaging system. Images were deconvolved using softWoRx ™ v3.0 (Applied Precision). Images were processed using Image J.

Live-cell imaging.

Live-cell imaging was performed on a Zeiss AxioObserver Z1 with a CSU-X1 spinning disc (Yokagowa), using a 63x/1.40 Plan-Apochromat lens and Evolve EMCCD camera (Photometrics) and motorised XYZ stage (ASI) driven by Marianas hardware and Slidebook 5.0 software (Intelligent Imaging Innovations). Cells were cultured on glass bottom 35 mm culture dishes (from MatTek and Iwaki).

FLIP: two regions of interest (ROI, marked in each figure) overlapping the cytosol and nucleus were bleached (two iterations, 488 nm, 100%, 50 ms laser duration). Photobleaching took approximately 30 seconds to cover both ROIs, and bleached both the cytoplasmic and nuclear fluorescence. Overlap with the nucleus was found to be important, as the size of GFP-Bax (approximately 50 kDa), is on the upper limit capable of diffusion through the nuclear pore complex (Paine et al., 1975). Thus, in the absence of bleaching the nuclear fraction, the slow diffusion of nuclear GFP-Bax into the cytosol over a period of 15-20 minutes was found to significantly impair imaging of loss of mitochondrial Bax.

Following photobleaching, images were captured every 30 seconds. For quantification of mitochondrial loss of fluorescence, images were analysed using Image J. Briefly, background fluorescence was subtracted (background was chosen as a ROI within the nucleus), cytosolic content exclusive of the bleached ROIs were selected and the signal decay quantified using ROI manager plugin in Image J. Data was normalised to 100% fluorescence post bleaching. Statistical analysis was carried out by ANOVA with Bonferroni's post-test (Prism, GraphPad). t ½ was calculated using non-linear regression analysis in GraphPad Prism.

Photoactivation: Cells were identified using mRFP-H2B, and photo-activated within one ROI (405 nm laser, 100%, 50 ms laser duration), which took approximately 15 seconds. Images were captured over 10 min at 30 s intervals. Mitochondrial association rate was determined using Image J in a similar way as for FLIP experiments. For photo-bleaching following photoactivation, a protocol similar to that used for FLIP was employed (two iterations, 488 nm, 100%, 50 ms laser duration), but which involved bleaching only within one ROI, as indicated in each figure. When imaging paGFP-Bax cells, we usually did not capture the mRFP-H2B signal, as this was only used to identify expressing cells. Figure 1E and G show images captured

specifically to illustrate mRFP-H2B expression, but for most image sequences used for quantification this channel was not captured.

FRAP: For FRAP, a single ROI was bleached (two iterations, 100%, 50 ms laser duration) as for the FLIP protocol, using 488 nm for YFP-BaxTMD and GFP-BclXL, and 561 nm for mRFP-BclXL. In cells co-expressing GFP-BclXL and DsRed-mito, GFP and DsRed were bleached simultaneously by the 488 nm laser, taking advantage of the multiple excitation peaks seen with DsRed. Quantification of fluorescence recovery was carried out in Image J using a similar protocol as for FLIP, measuring within the bleached ROI.

Subcellular fractionation and western blotting.

Subcellular fractionation and membrane integration assays were carried out as previously described (Valentijn et al., 2008). Cells were transfected with either pCDH-GFP-Bax or pCDH-GFP-BaxS184V. 18hr post-transfection cells were scraped into hypotonic lysis buffer (10mM Tris.Cl, pH 7.5, 10mM NaCl,1.5 mM MgCl2) containing protease inhibitors and allowed to swell on ice for 10 min prior to homogenising with a glass dounce homogeniser. MS buffer (2.5X; 525 mM mannitol,175mM sucrose, 12.5 mM Tris.Cl, pH 7.5, 2.5 mM EDTA) was added to 1X. The homogenates were centrifuged at 1300g for 10 min at 4°C. The supernatant was centrifuged at 17000g for 15 min at 4°C to obtain the mitochondrial pellet, which was then washed once with 1X MS buffer and then solubilised in 0.1M Na_2CO_3 for 30 min on ice. After centrifugation at 100,000g for 30 min at 4°C, the supernatant (alkali-soluble fraction) and pellet (alkali-resistant fraction) were analyzed by Western blotting.

Western blotting was performed by lysing cells in RIPA buffer (50 mM Tris-HCl pH 7.5, 150mM NaCl, 1% NP40, 0.5 % sodium deoxycholate, 1 mM EDTA, 100 µM Na_3VO_4, 10 mM NaF, protease inhibitor cocktail). Lysates were separated by SDS-PAGE, transferred to nitrocellulose membranes and immunoblotted.

Supplemental References

Campbell, R.E., Tour, O., Palmer, A.E., Steinbach, P.A., Baird, G.S., Zacharias, D.A., and Tsien, R.Y. (2002). A monomeric red fluorescent protein. Proc Natl Acad Sci U S A *99*, 7877-7882.

Gilmore, A.P., Metcalfe, A.M., Romer, L.H., and Streuli, C.H. (2000). Integrin-mediated survival signals regulate the apoptotic function of Bax through its conformation and subcellular localization. J Cell Biol *149*, 431-445.

Kittrell, F.S., Oborn, C.J., and Medina, D. (1992). Development Of Mammary Preneoplasias Invivo From Mouse Mammary Epithelial-Cell Lines Invitro. Cancer Res *52*, 1924-1932.

Littlewood, T.D., Hancock, D.C., Danielian, P.S., Parker, M.G., and Evan, G.I. (1995). A modified oestrogen receptor ligand-binding domain as an improved switch for the regulation of heterologous proteins. Nucleic Acids Res *23*, 1686-1690.

Paine, P.L., Moore, L.C., and Horowitz, S.B. (1975). Nuclear envelope permeability. Nature *254*, 109-114.

Patterson, G.H., and Lippincott-Schwartz, J. (2002). A photoactivatable GFP for selective photolabeling of proteins and cells. Science *297*, 1873-1877.

Valentijn, A.J., Metcalfe, A.D., Kott, J., Streuli, C.H., and Gilmore, A.P. (2003). Spatial and temporal changes in Bax subcellular localization during anoikis. J Cell Biol *162*, 599-612.

Valentijn, A.J., Upton, J.P., Bates, N., and Gilmore, A.P. (2008). Bax targeting to mitochondria occurs via both tail anchor-dependent and -independent mechanisms. Cell Death Differ *15*, 1243-1254.
