# Supplementary figures and images for: Bax Exists in a Dynamic Equilibrium between the Cytosol and Mitochondria to Control Apoptotic Priming
Source: Mol Cell. 2013 Mar 7;49(5):959–71. doi: 10.1016/j.molcel.2012.12.022 (PMC3594749; doi:10.1016/j.molcel.2012.12.022)

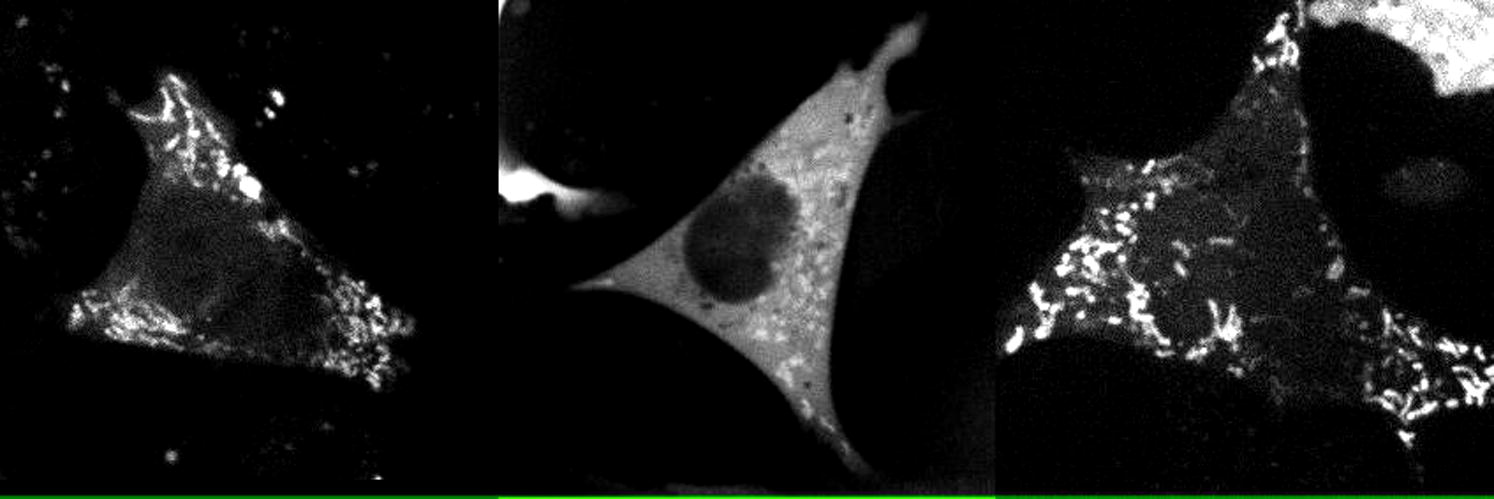

Supplement: Movie S2. FLIP Analysis of GFP-Bax and GFP-BaxS184V in Bax−/−Bak−/− DKO MEFs, Related to Figure 1 — Movie S2 is related to Figure 1C and shows (left to right) Bax−/−Bak−/− DKO MEFs transfected with GFP-Bax, GFP-Bax treated with ABT-737, and GFP-BaxS184V. [file mmc3.jpg]

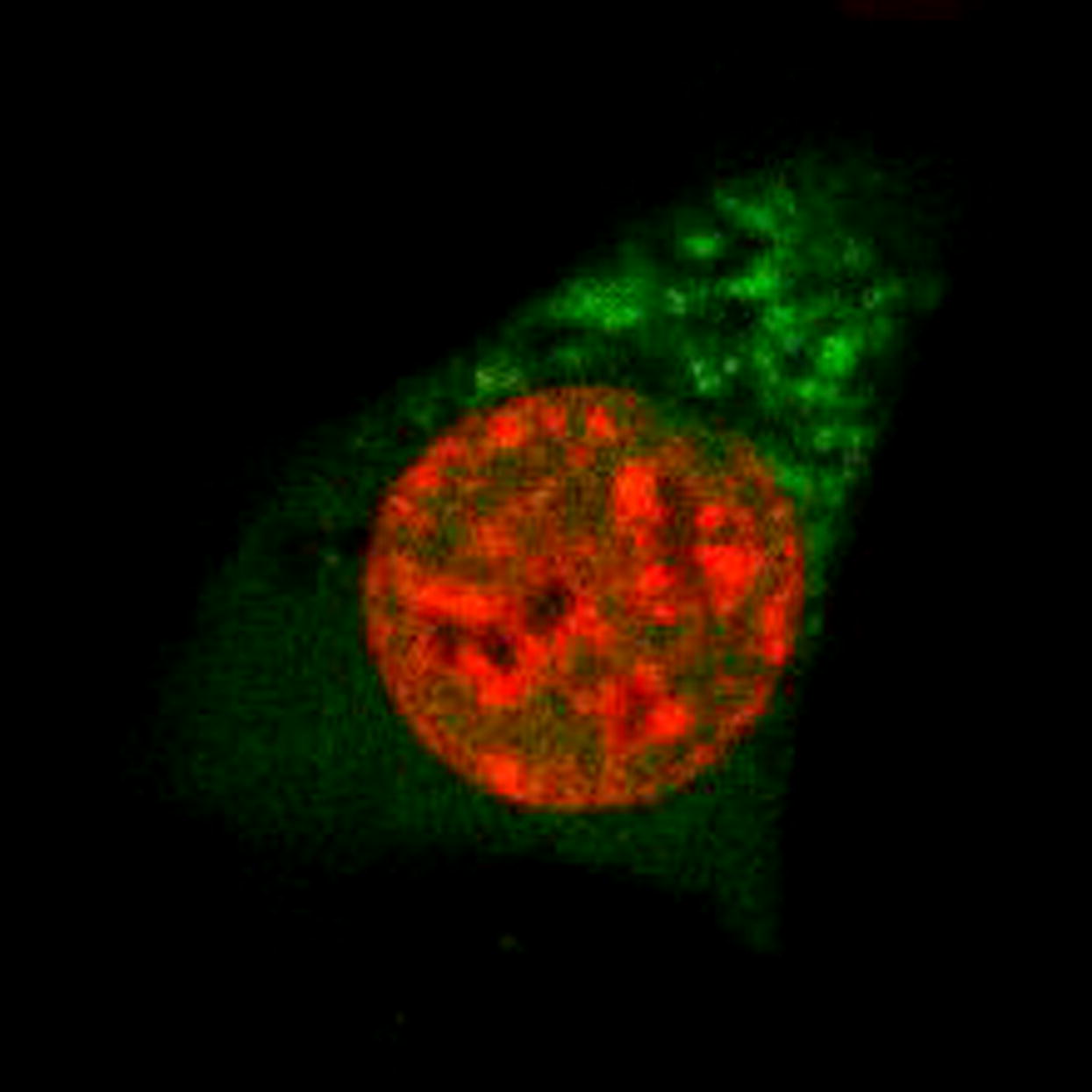

Supplement: Movie S4. Photoactivation of paGFP-Bax Expressed in MECs, Related to Figure 2 — Movie S4 relates to Figure 2A, and shows the accumulation of paGFP-BaxS184V on mitochondria. The cell also shows the coexpressed mRFP-H2B. [file mmc5.jpg]
